# Dissecting the Shared and Context-Dependent Pathways Mediated by the p140Cap Adaptor Protein in Cancer and in Neurons

**DOI:** 10.3389/fcell.2019.00222

**Published:** 2019-10-15

**Authors:** Jennifer Chapelle, Oksana Sorokina, Colin McLean, Vincenzo Salemme, Annalisa Alfieri, Costanza Angelini, Alessandro Morellato, Annie Adrait, Elisabetta Menna, Michela Matteoli, Yohann Couté, Ugo Ala, Emilia Turco, Paola Defilippi, J. Douglas Armstrong

**Affiliations:** ^1^Department of Molecular Biotechnology and Health Sciences, Università degli Studi di Torino, Turin, Italy; ^2^Simons Initiative for the Developing Brain, School of Informatics, The University of Edinburgh, Edinburgh, United Kingdom; ^3^Univ. Grenoble Alpes, CEA, INSERM, IRIG, BGE, Grenoble, France; ^4^Institute of Neuroscience, CNR, Milan, Italy; ^5^Istituto Clinico Humanitas, IRCCS, Rozzano, Italy; ^6^Department of Veterinary Sciences, Università degli Studi di Torino, Turin, Italy

**Keywords:** p140Cap, SRCIN1, mass spectrometry, protein interaction network, breast cancer, neuronal synapses

## Abstract

The p140Cap adaptor protein is a scaffold molecule physiologically expressed in few epithelial tissues, such as the mammary gland, and in differentiated neurons. While the role of p140Cap in mammary gland epithelia is not still understood, we already know that a significant subset of breast cancers express p140Cap. In the subgroup of ERBB2-amplified breast cancers, a high p140Cap status predicts a significantly lower probability of developing a distant event and a clear difference in survival. p140Cap is causal in dampening ERBB2-positive tumor cell progression, impairing tumor onset and growth, and counteracting epithelial mesenchymal transition, resulting in decreased metastasis formation. Since only a few p140Cap interacting proteins have been identified in breast cancer and the molecular complexes and pathways underlying the cancer function of p140Cap are largely unknown, we generated a p140Cap interactome from ERBB2-positive breast cancer cells, identifying cancer specific components and those shared with the synaptic interactome. We identified 373 interacting proteins in cancer cells, including those with functions relevant to cell adhesion, protein homeostasis, regulation of cell cycle and apoptosis, which are frequently deregulated in cancer. Within the interactome, we identified 15 communities (clusters) with topology-functional relationships. In neurons, where p140Cap is key in regulating synaptogenesis, synaptic transmission and synaptic plasticity, it establishes an extensive interactome with proteins that cluster to sub complexes located in the postsynaptic density. p140Cap interactors converge on key synaptic processes, including synaptic transmission, actin cytoskeleton remodeling and cell-cell junction organization. Comparing the breast cancer to the synaptic interactome, we found 39 overlapping proteins, a relatively small overlap. However, cell adhesion and remodeling of actin cytoskeleton clearly emerge as common terms in the shared subset. Thus, the functional signature of the two interactomes is primarily determined by organ/tissue and functional specificity, while the overlap provides a list of shared functional terms, which might be linked to both cancer and neurological functions.

## Introduction

p140Cap/SNIP (Chin et al., 2000; Di Stefano et al., 2004) is a scaffolding protein encoded by the *SRCIN1* gene, and is localized in epithelial tissues (Damiano et al., 2010), such as the mammary gland, and in dendritic spines (Jaworski et al., 2009). In the normal human breast, p140Cap is expressed selectively in luminal cells of alveoli, whereas no staining is detectable in ductal epithelial cells or myoepithelial cells (Damiano et al., 2010). Although its role in the mammary gland is not yet well established, an oncosuppressive role for p140Cap in breast cancer has been already proven. p140Cap immunohistochemistry (IHC) on a large cohort of invasive breast cancers indicate that positive p140Cap status was associated with good prognosis markers, such as negative lymph node status, estrogen and progesterone receptor-positive status, small tumor size, low grade, and low proliferative status. Positive p140Cap status was also associated to breast cancer molecular subtypes, being expressed in >85% of Luminal A tumors, 77% of Luminal B, and only 56% of triple-negative tumors (Grasso et al., 2017). In patients with ERBB2-amplified breast cancer, a p140Cap-positive status associates with a significantly lower probability of developing a distant event, and a clear difference in survival (Grasso et al., 2017). A well-characterized model of ERBB2-dependent breast carcinogenesis is the NeuT mouse (Muller et al., 1988; Boggio et al., 1998). The NeuT endogenous tumors do not express p140Cap, thus representing a patient with low or undetectable expression of p140Cap. We have already generated a transgenic mouse model in which p140Cap is specifically expressed in the mammary gland, and we crossed these mice with the NeuT mice. Consistent with the data obtained in the human breast cancer cohort, the double transgenic mice p140Cap-NeuT attenuates the phenotype of NeuT tumors *in vivo*, resulting in the development of smaller and lower grade mammary carcinomas (Grasso et al., 2017). Moreover, we also set-up an additional, transplantable primary model, the NeuT-TUBO (Rovero et al., 2000). Consistent with the transgenic model, the lack of p140Cap expression in these cells renders them suitable to address whether p140Cap gain of function may affect tumorigenic phenotype. Indeed, p140-TUBO cells limits tumor cell growth upon transplantation, with a significantly reduced number of spontaneous lung metastases (Grasso et al., 2017). Overall, p140Cap dampens ERBB2-positive tumor cell progression, impairing tumor onset and growth, and counteracting epithelial mesenchymal transition, resulting in decreased metastasis formation (Di Stefano et al., 2007; Cabodi et al., 2010; Grasso et al., 2017).

The specific role of p140Cap in curbing the aggressiveness of ERBB2-amplified breast cancers may rely on its ability to impinge on specific molecular pathways. Amongst the functions of the p140Cap adaptor, is its ability to bind and regulate Src kinase activation, shifting the balance of active to inactive Src (Di Stefano et al., 2007). p140Cap impairs adhesion-dependent integrin signaling (Di Stefano et al., 2007), as well as E-cadherin dependent cell-cell adhesion, which results in a suppression of the scattering properties of breast and colon cancer cells (Damiano et al., 2010). The ability to down-regulate Src kinase activity was also observed in physiological conditions, in crude mouse synaptosomal fractions (Repetto et al., 2014), indicating that this pathway is common to both cancer cells and neurons. Interestingly, in ERBB2 transformed cells, p140Cap exerts a suppressive function on migratory and invasive features, with a negative regulatory impact on the molecular pathways that ERBB2 exploits for tumor progression, such as the Tiam1/Rac GTPase axis (Grasso et al., 2017).

Previous work from our laboratory and others indicates that in neurons, in physiological conditions, p140Cap has a key role in regulating synaptogenesis, synaptic transmission and synaptic plasticity (Jaworski et al., 2009; Tomasoni et al., 2013; Repetto et al., 2014). Acute down-regulation of p140Cap in primary hippocampal neurons reduces the number of mushroom spines and proportionally increases the number of dendritic filopodia (Jaworski et al., 2009; Tomasoni et al., 2013); a defect in synaptic maturation that can also be observed in p140Cap knockout (KO) mice (Repetto et al., 2014).

The assembly of multi-protein complexes (interactomes) is key for triggering signaling mechanisms key for the execution of basic biological functions. Several examples come from the assembly of signaling complexes regulating cell migration and proliferation, in which PPIs are built around adaptor proteins. These complexes may localize at either the plasma membrane level, bringing membrane receptors into close proximity of cellular components, or in the cytoplasm, or in specific organelles. Molecular interactomes in cells and tissues may be interrogated using mass spectrometry (MS) combined with bioinformatics data and analyses. To study protein complexes that underlie cell organization and its functions, the data from interactome studies is often represented via static undirected PPI Networks. Clustering algorithms and parameters can be used to identify heterogeneous communities within the network, which share topological properties. These communities often form “modules” of proteins that functionally co-operate in specific pathways. Gene-disease and gene-functional annotation data can then be annotated onto those clusters to test functional/disease enrichment of the clusters. This can be used to predict new candidate genes to be associated with known diseases (Mclean et al., 2016).

Although p140Cap has been shown to recruit and regulate specific signaling molecules both in breast cancer cells and in healthy neuronal synapses, the molecular complexes and pathways underlying p140Cap function in pathological and physiological conditions are largely unknown. Recently, in a neuronal context, we reported 351 p140Cap interacting proteins that were isolated by co-immuno precipitation from mouse synaptosomes (Alfieri et al., 2017). We showed that those proteins were involved in key synaptic processes, including transmission across chemical synapses, actin cytoskeleton remodeling and cell-cell junction organization. Furthermore, we found strong association of those proteins with neurological diseases, such as schizophrenia, autism, bipolar disorder, intellectual disability, and epilepsy.

Here we exploited the transplantable primary NeuT cell model, NeuT-TUBO and p140-TUBO cells (Grasso et al., 2017), to capture the p140Cap molecular complexes and to pinpoint interactions crucial for regulation of ERBB2-positive cancer-specific features. Using biochemical and proteomic data, and bioinformatics tools, we were able to provide a first comprehensive analysis of the specific p140Cap PPI network in NeuT/ERBB2 breast cancer cells.

Even though cancer cells and neurons are quite different, there is growing evidence that metastatic cancer cells could implement signaling mechanisms common to those used in the homeostasis of synaptic growth/plasticity (Heine et al., 2015). We then compared the p140Cap cancer interactome with the synaptic one, revealing that p140Cap does participate in some common pathways in the two distinct cellular contexts, which may underlie shared biological mechanisms between neurons and tumor cells. To our knowledge this is one of the first examples of an adaptor protein that participates to biological complexes that are either specific for organs and tissues, or overlapping to both cancer and neurological functions.

## Materials and Methods

### Isolation, Identification, and Validation of p140Cap Interactome in Cancer Cells

#### ERBB2 Breast Cancer Cell Model

TUBO cells are a transplantable primary breast cancer NeuT cell model from the BALB/c background. Upon infection with empty or p140Cap retroviruses, we generated NeuT-TUBO (as control cells), and p140-NeuT-TUBO cells, as described in Grasso et al. (2017) (hereafter called mock and p140 cells). Mock and p140 cells were cultured in DMEM 20% FBS. Culture media were from Invitrogen (Carlsbad, CA, United States). Fetal Calf serum (FCS) was from EuroClone (Pero, Milano, Italy).

#### p140Cap Immunoprecipitation

p140Cap antibodies were cross-linked to protein G Dynabeads (Invitrogen, Carlsbad, CA, United States) as described in Alfieri et al. (2017). p140Cap Mab-coupled Dynabeads were incubated with 9 mg of cell extracts from NeuT-TUBO and p140-TUBO cells, grown at 80% confluency and extracted with Lysis buffer (150 mM NaCl, 50 mM Tris pH 7.4, 1% NP-40, 1 mM MgCl_2_. 5% glycerol) for 2 h at 4°C. Beads were washed five times with cold lysis buffer, then resuspended in 45 μl of 2% SDS-PAGE sample buffer in reducing conditions and incubated at 70°C for 10 min. From this 1/9 of the sample was used for Coomassie staining, 1/9 for Western blot analysis of p140Cap to assess the quality of the immunoprecipitation, and 7/9 was used for MS analysis.

#### Mass Spectrometry-Based Proteomic Analyses

IPs eluate proteins were stacked in the top of a SDS-PAGE gel to be able to treat the whole sample in a single band, and in-gel digested using modified trypsin (Promega, sequencing grade) as previously described (Alfieri et al., 2017). Resulting peptides were analyzed by online nanoLC-MS/MS (UltiMate 3000 and LTQ-Orbitrap Velos Pro, Thermo Scientific). For this, peptides were sampled on a 300 μm × 5 mm PepMap C18 precolumn and separated on a 75 μm × 250 mm C18 column (PepMap, Thermo Scientific). MS and MS/MS data were acquired using Xcalibur (Thermo Scientific). Peptides and proteins were identified and quantified using MaxQuant, version 1.5.8.3 (Tyanova et al., 2016). Spectra were searched against the Uniprot database (*Mus musculus* taxonomy, May 2017 version) and the frequently observed contaminants database embedded in MaxQuant. Trypsin was chosen as the enzyme and two missed cleavages were allowed. Peptide modifications allowed during the search were: carbamidomethylation (C, fixed), acetyl (Protein N-ter, variable) and oxidation (M, variable). Minimum peptide length was set to seven amino acids. Minimum number of peptides, razor + unique peptides and unique peptides were all set to 1. Maximum false discovery rates (FDR) - calculated by employing a reverse database strategy - were set to 0.01 at peptide and protein levels. The matching between runs option was activated. The MS proteomics data have been deposited to the ProteomeXchange Consortium via the PRIDE partner repository (Vizcaino et al., 2016) with the dataset identifier PXD008778.

Statistical analysis was performed using ProStaR (Wieczorek et al., 2017). In this protocol, iBAQ values were used to be able to compare those results with those previously obtained on p140Cap interactome in synaptosome (Alfieri et al., 2017). Proteins identified in the reverse and contaminant databases, proteins only identified by site, proteins identified with only 1 peptide and proteins exhibiting less than 3 iBAQ values in one condition were discarded from the list. After log2 transformation, iBAQ values were normalized by median centering before missing value imputation (replacing missing values by the 2.5 percentile value of each column); statistical testing was conducted using limma *t*-test. Differentially interacting proteins were sorted out using a log2 (fold change) cut-off of 1 and a FDR threshold on remaining *p*-values of 1% using the Benjamini-Hochberg method.

#### Antibodies

Specific mouse monoclonal antibody (Mab) against p140Cap (clone 2A8) was produced at the MBC, University of Torino, as previously described (Di Stefano et al., 2007; Repetto et al., 2013). The antibodies used are as follow: mouse monoclonal antibodies to anti p140Cap (1:500), anti E-Cadherin (1:1000), anti Erbb2 (1:1000), anti Vimentin (1:1000) and polyclonal rabbit anti Skt (1:1000) from the Antibody production facility of the Dept of Molecular Biotechnology and Health Sciences, University of Torino; anti α-Catenin (#3236, 1:1000), anti δ-Catenin (#34989, 1:1000), anti TECPR1 (#8097, 1:1000) from Cell Signaling, Beverly, MA, United States; anti Iqgap1 (sc-10792, 1:500) from Santa Cruz Biotechnology, Palo Alto, CA, United States; anti β-Catenin (cat.no 6101531, 1:1000) from BD transduction Laboratories, Franklin Lakes, NY, United States; anti Talin (ab11188, 1:1000) from Abcam, Cambridge, United Kingdom. Mouse and Rabbit IgGs were purchased from Santa Cruz Biotechnology. Secondaries antibodies anti-mouse and anti-rabbit were purchased from Sigma-Aldrich Co., Italy.

#### Western Blot

Western blots were performed with Mini-PROTEAN^®^ TGX^TM^ Precast Gels from Bio-Rad (California 94547 United States) gradient 4–15% Gels were transferred onto Nitrocellulose blotting membrane (GE Healthcare Life Sciences) using Towbin buffer (25 mM Tris, 192 mM Glycine, 20% Methanol). Membranes were blocked with Tris–buffered saline TBS (50 mM Tris ph7–150 mM NaCl) with 5% Milk for 1 h at room temperature, incubated with primary and secondary antibodies as indicated below, and then developed with Bio-Rad’s Clarity ECL on ChemiDoc Touch Imaging System (Biorad). For Western blot, 30 ug of protein extract were used.

## Bioinformatic Analyses of MS Data

### Building the Protein-Protein Interaction Network and Clustering

p140Cap networks were constructed from 373 proteins for the cancer interactome and from the previously published 351 proteins for the synaptic interactome. Protein-protein interactions were obtained by mining publicly available databases: BioGRID (Chatr-Aryamontri et al., 2015), IntAct (Kerrien et al., 2012) and DIP (Salwinski et al., 2004). The first two are gold standard PPI repositories, and were used together with DIP because they are defined to the same standardized format (i.e., PSI-MI), which provides a way to filter for a set of “direct and physical” human interactions obtained in experiment - interactions not predicted or inferred. Our set of PPIs was constructed then, by selecting only those MI Ontology terms which are related to “direct and physical” interactions. We also included interactions (in our set) from BioPLEX, since the BioPlex PPIs are already deposited in the Intact and BioGRID databases. The largest connected component of each network was split into a set of communities by use of five clustering algorithms. Those included the Modularity –maximization based algorithms: agglomerative random walk (wt) (Pons and Latapy, 2006), the coupled Potts/Simulated Annealing “SpinGlass”(sg) (Reichardt and Bornholdt, 2006; Traag and Bruggeman, 2009), and the divisive spectral based fine tuning (Spectral) (Mclean et al., 2016), and Non-Modularity based algorithms, including information-theoretic based “InfoMAP” algorithm (infomap) and the Mixed-Membership Stochastic Blockmodel “SVI” (Gopalan and Blei, 2013).

### Function and Disease Enrichment and Annotation

Throughout this study, overrepresentation of annotation terms (disease, function, etc.) was estimated by use of the hypergeometric distribution to test whether the number of selected proteins is larger than would be expected by chance:

$$p=1-\sum_{i=0}^{k-1}\frac{\left(\frac{M}{i}\right)\,\left(\frac{N-M}{n-i}\right)}{\left(\frac{N}{n}\right)},$$

where N is a total number of proteins in the background distribution, M is the number of genes within distribution that are annotated to the term of interest, n is the size of the list of genes of interest and k is the number of genes within the list, which are annotated to the term. Obtained *p*-values were adjusted for multiple testing by Bonferroni correction at 0.05 (^∗^), 0.01 (^∗∗^) and 0.001 (^∗∗∗^).

Enrichment analysis for functional annotations in the interactome was performed in R, using the Bioconductor packages ClusterProfiler for Gene Ontology (GO) and KEGG enrichment analysis (Yu et al., 2012) and Reactome PA for pathway over-representation analysis. The default mouse genome list from Bioconductor was used as a background set. *P*-values, adjusted for multiple comparison *p*.adjust and *q*-values for FDR are provided in Supplementary Table S2.

For disease enrichment the annotation data were standardized using MetaMap (Aronson and Lang, 2010) and NCBO Annotator (Whetzel et al., 2011; Musen et al., 2012) to recognize terms found in the Human Disease Ontology (HDO) (Schriml et al., 2012). We focused on the following disease list (Table 3) trying to cover well known relevant pathologies as follows.

For neurological conditions: Schizophrenia (SCH), Alzheimer’s disease (AD), Autistic Spectrum Disorder (ASD), Autistic Disorder (AUT), Bipolar Disorder (BD), Epilepsy Syndrome (Epi), Temporal Lobe Epilepsy (TLE), Focal Epilepsy (Fepi), Parkinson’s Disease (PD), Frontotemporal Dementia (FTD), Huntington’s Disease (HD) and Intellectual Disability (ID).

For cancer: Neuroblastoma (NB), Autonomic Nervous System Neoplasm (ANSN), Peripheral Nervous System Neoplasm (PNSN), Nervous System Cancer (NSC), Central Nervous System cancer (CNSC), Malignant Glioma (MG), Stomach cancer (SC). Gastrointestinal System cancer (GISC), Stomach carcinoma (SCA), Gastric Adenocarcinoma (GAC), Gastric Lymphoma (GLC), Breast cancer (BC), Melanoma (MEL), Hepatocellular carcinoma (HCC), Squamous cell carcinoma (SCC).

Enriched disease ontology terms were then associated with protein identifiers and the associations stored locally. Enrichment of disease terms was then calculated using the Topology-based Elimination Fisher method (Alexa et al., 2006) found in the topGO package, together with the standardized OMIM and Ensembl variation gene-disease annotation data mapped onto the full HDO tree.

The significance of annotation enrichment in each cluster was tested by Hypergeometric distribution. Enriched association with *P* ≤ 0.01 were further tested for their strength of significance by recording the percentage of *P*-values found from every community/annotation combination, lower than or equal to the observed *P*-value, when 1,000 random permutations of the annotation labels were made. *P*-values found with strength of significance, 1% were considered statistically significant. *P*-values were also tested against a more stringent Bonferroni correction at 0.05 (^∗^), 0.01 (^∗∗^) and 0.001 (^∗∗∗^) significant levels, and highlighted throughout enrichment tables.

### Identifying the Influential Proteins in the Network

For concrete clustering algorithms we made use of the boot-strap procedure (Simpson et al., 2010) and vertex degree to calculate the vertex’s community membership: the approach is similar in spirit to the use of within module degree and the participation coefficient in classify the biological importance of proteins in Metabolic networks (Guimera and Nunes Amaral, 2005).

To identify influential genes using the topology of the network, we made use of the semi-local centrality measure *Cl(v)* of a vertex *v* (Chen et al., 2010). Semi-local centrality measure takes into consideration both a vertex’s degree, its nearest, and next to nearest neighbors:

$$Q\,\left(u\right)=\sum_{w\in \Gamma \,\left(w\right)}N\,\left(w\right)$$

$$C\,l\,\left(v\right)=\sum_{v\in \Gamma \,\left(v\right)}Q\,\left(u\right),$$

where *Γ(u)* is the set of nearest neighbors of *u* and *N(w)* the number of nearest and next to nearest neighbors of vertex *w*. We performed unity-based normalization to bring the semi-local centrality values into the range [0,1].

To measure the influence of a gene due to the clustering algorithm *a* we make use of Bridgeness *Ba(v)* of a vertex *v* (Nepusz et al., 2008)

$$B\,a\,\left(v\right)=1-\sqrt{\frac{c}{c-1}\,\sum_{j=1}^{c}{\left(u_{j\,v}-\frac{1}{c}\right)}^{2}}$$

Here *u* is the community membership of vertex *v*, that is the probability of vertex *v* to belong to a given community:

$$u_{j\,v}=\left[u_{1\,v},u_{2\,v},\ldots ,u_{c\,v}\right],$$

where ∑_*v,j*_ *u*_*jv*_ = 1, and *c* is the number of communities detected by algorithm. To classify each protein we took the average Bridging score across each algorithm:

$$B\,r\,\left(v\right)=\frac{1}{N}\,\sum_{a\in A\,\mathrm{lg}}^{N}B\,a\,\left(v\right),$$

where *Alg* is the set of clustering algorithms, i.e., sg, Spectral, infomap, and SVI. The Bridgeness measure lies between 0, implying a vertex belongs to a single community, and 1, implying a vertex forms a “global bridge” across every community with the same strength (see section “Materials and Methods” for details). By plotting Bridgeness against semi-local centrality we can categorize the influence each proteins has on network structure.

Plotting Bridgeness against local centrality allows us to partition the proteins into four quadrants, or regions, labeled 1–4. Results of analysis for consensus clustering is shown on the Figure 6:

1) Bridging proteins with “global” rather than “local” influence (also been called bottle-neck bridges (Najafi et al., 2016), connector or kinless hubs (Guimera and Nunes Amaral, 2005), lie in the range 0 ≤ Cl ≤ 0.5 and 0.5 ≤ Br ≤ 1 (Region 1, Figure 5).
2) Bridging proteins with mixed ‘global’ and ‘local’ influence in the network, lie in the range 0.5 ≤ Cl ≤ 1 and 0.5 ≤ Br ≤ 1 (Region 2, Figure 5).
3) Proteins important primarily within one or two communities (local or party hubs (Nepusz et al., 2008), lie in the ranges 0 ≤ Cl ≤ 0.5 and 0.1 ≤ Br ≤ 0.5 (Region 3, Figure 5).
4) Proteins that influence just “locally” in the network in the range 0.5 ≤ Cl ≤ 1.0 and 0.0 ≤ Br ≤ 0.5 (Region 4, Figure 5).

Due to disassortative mixing, i.e., a preference for high degree proteins to attach to low-degree proteins, most of the proteins have 0 ≤ Br ≤ 0.1, i.e., too small to have any effect on the networks complexes (Region 4). In our study we define the “bridging” proteins as those found Regions 1 and 2.

### Estimating the Overlap Between Diseases Annotation

We tested our gene-disease annotation (GDA) data on p140Cap synaptic and Cancer PPI networks, using a network-based approach to identify the location of disease modules, localized regions of connections between disease-related proteins, on the interactome (Menche et al., 2015). We investigated the overlap and separation of each disease-disease pair by measuring the mean shortest distance for each disease *d*, using the shortest distance between each GDA to its next nearest GDA neighbor. The overlap, or separation, of each disease-disease pair in the network could be quantified using:

$$S_{A\,B}=d_{A\,B}-\frac{d_{A\,A}-d_{B\,B}}{2}$$

where *d*_*AA*_ and *d*_*BB*_ quantify the mean shortest distances within the respective diseases and *d*_*AB*_- the mean shortest distances between diseases. *S*_*AB*_ is bound by the diameter of the network., i.e., *d*_max_ ≤ *S*_*AB*_ ≤ *d*_max_, where *d*_max_ is 8 for synaptic and 6 for cancer networks, respectively. The magnitude of *S*_*AB*_ depends on the number of GDAs associated with each disease. Large positive values imply two well separated diseases, while large negative values indicate large (i.e., number of GDAs) diseases with a substantial overlap, often implying that one disease is the variant or precursor to the other. In general disease-disease pairs with *S*_*AB*_ < −3 or *S*_*AB*_ > 0.1 were considered of interest. Each disease-disease pairs observed *S*_*AB*_ value was tested computed against a full-randomized model: drawing the same number of GDAs (from the set of all network genes) for each disease at random, before computing its separation$S_{A\,B}^{r\,a\,n\,d}$. For each disease-disease pair, we performed 1000 iterations of the full randomized model.

The difference between the observed and randomized disease pair separation was quantified using the Z-score:

$$z-s\,c\,o\,r\,e=\frac{S_{A\,B}-\left(S_{A\,B}^{r\,a\,n\,d}\right)}{\sigma \,\left(S_{A\,B}^{r\,a\,n\,d}\right)}$$

Where $S_{A\,B}^{r\,a\,n\,d}$ and $\sigma_{A\,B}^{r\,a\,n\,d}$ are the random disease-disease pair mean and standard deviation obtained from 1000 iterations. Negative (positive) z-scores imply that the disease-disease separation or overlap is smaller (larger) than expected by chance. To assess the significance of each disease-disease pairs overlap or separation, *P*-values were estimated based on the z-scores above, and tested against the stringent Bonferroni correction at the 0.05 (^∗^), 0.01 (^∗∗^) and 0.001 (^∗∗∗^) significance levels.

## Results

### Quantitative Proteomic Analysis of p140Cap Cancer Interactome

**FIGURE 1:**
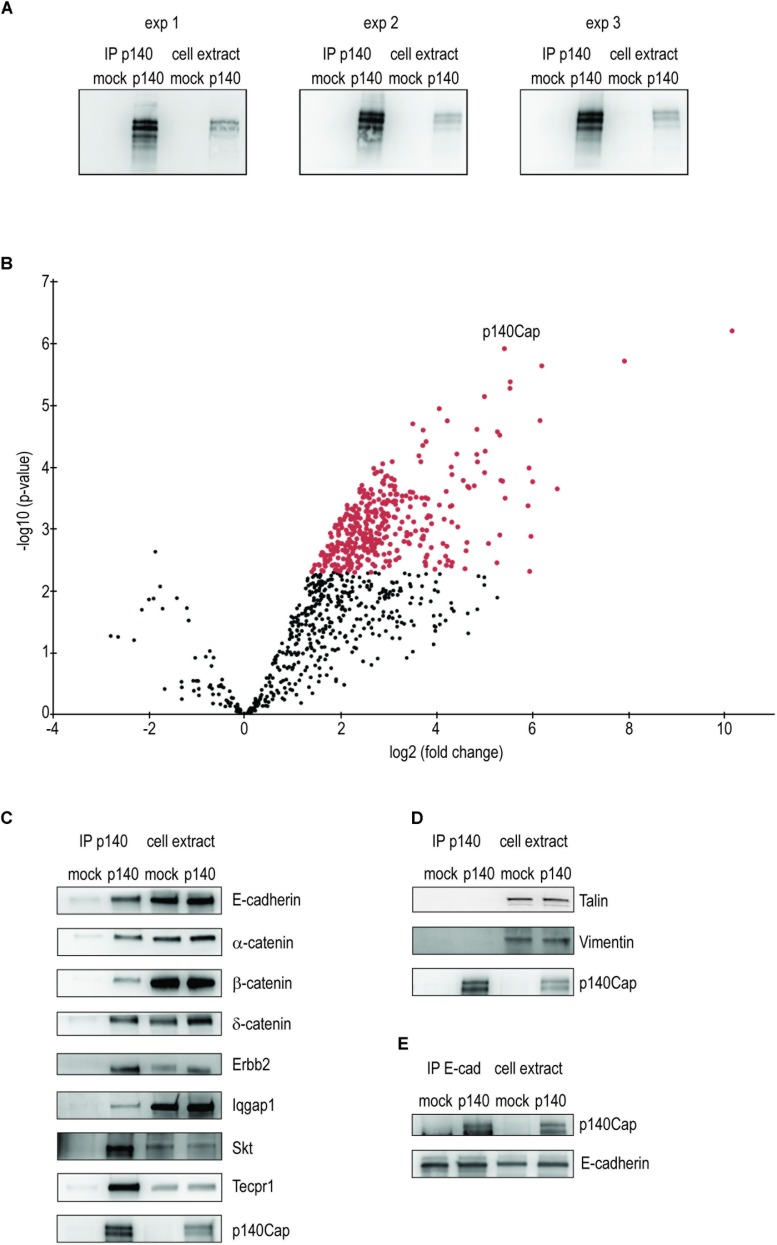
Identification and validation of p140Cap interactors in p140Cap-TUBO breast cancer cells. Identification and validation of p140Cap interactors. **(A)** Validation of the breast cancer cell extracts. Western blot for p140Cap from the three immunoprecipitation of p140Cap from Erbb2 TUBO murine breast cancer cells. **(B)** Statistically enriched proteins in the p140Cap IP. The Volcano plot represents the log10 (*p*-value, *y* axis) plotted against the log2 (fold change, *x* axis) for proteins quantified in p140Cap IPs from mock and p140Cap KO, used as negative control. 373 different proteins, including p140Cap (arrowhead) were found significantly enriched in p140 samples (FDR ≤ 1%, ≥ 4-fold enrichment) are shown as red dots. **(C)** Validation of synaptic p140Cap interacting proteins identified in the interactome. Co-immunoprecipitated proteins are shown on the left. **(D)** Negative controls by Western blot of Talin and Vimentin. **(E)** Reverse validation for p140Cap interacting protein E-Cadherin.

We have already shown that upon transplantation in syngeneic mice, p140-NeuT-TUBO cell-derived tumors showed significantly limited growth and metastasis formation over tumors derived from implanted NeuT-TUBO cells (Grasso et al., 2017), demonstrating that in this breast cancer model, p140Cap is sufficient “*per se*” to impair *in vivo* tumor progression. Therefore we selected this cellular model to analyze and characterize the p140Cap interactome, in order to uncover protein complexes and the embedded functional pathways to which p140Cap may associate in breast cancer. p140-NeuT-TUBO cells in cell culture show a significant defect in cell proliferation associated to a reduced colony size in an anchorage-independent assay (Supplementary Figure S1), indicating that p140Cap controls tumor growth also in *in vitro* conditions. Therefore, we performed quantitative proteomic analysis of p140Cap immunoprecipitates from p140-NeuT-TUBO cells, using NeuT-TUBO (Grasso et al., 2017) negative control (hereafter called p140 and mock cells). Proteins were immunoprecipitated from both cell types at 80% confluence with the p140Cap monoclonal antibody and three separate experiments were performed. A p140Cap immunoreactive band was observed in the p140Cap IPs from p140 cells but not from mock extracts (Figure 1A and Supplementary Figure S2), confirming that p140Cap is present only in p140 cells, thus making these immunoprecipitates suitable for the identification of p140Cap interactors by MS over an empty control.

To identify p140Cap-binding partners, we applied label-free quantitative MS-based proteomics to the p140Cap IPs from p140 and mock cells. Proteins eluted from the IPs were stacked in the top of a SDS-PAGE gel to be able to treat the whole sample in a single band and in-gel digested. The resulting peptides were analyzed by nanoliquid chromatography coupled to tandem MS. Stringent statistical analysis allowed us to identify 374 (373 interactors plus p140Cap) proteins enriched in the p140 samples (Supplementary Table S1), as represented in the Volcano plot (Figure 1B). Differentially interacting proteins were classified using a log2 (fold change) cut-off of 1 and a fold-discovery-rate (FDR) threshold on remaining *p*-values of 1% using the Benjamini-Hochberg method.

To validate the proteomic findings, we selected 8 candidate interactors of p140Cap, performing Western blots with specific antibodies on p140Cap immunoprecipitates from p140 and mock cells. We validated the first one in the list (see Supplementary Table S1), Tecpr1, alias Tectonin beta-propeller repeat-containing protein 1, involved in autophagy (Chen and Zhong, 2012); in addition we tested Cadherin-1, Catenin beta-1, Catenin alpha-1 and Catenin delta-1, all involved in epithelia cell-cell interaction. We also validated the NeuT/Erbb2 oncogene and the Ras GTPase-activating-like protein IQGap1 (Hedman et al., 2015). SKT (Sickle tail protein), already found in the synaptic interactome (Alfieri et al., 2017) was also validated. The Western blots are shown in Figure 1C. Cadherin-1 and Catenin beta-1 have been already shown to interact with p140Cap in human breast cancer MCF7 cells (Damiano et al., 2010). We also verified that Talin and Vimentin, placed low in the interactome, were not immunoprecipitated with anti p140Cap Mab (Figure 1D). Further, we performed a reverse validation: we immunoprecipitated E-cadherin and verified the presence of p140Cap (Figure 1E). The validation of the proteomic data across a range of various enrichment levels above the fixed cut-off gives us confidence that the p140Cap cancer interactome we isolated contains bona fine p140Cap-interacting macromolecular complexes.

### Functional Characterization of the p140Cap-Containing Protein Complex in Breast Cancer

**FIGURE 2:**
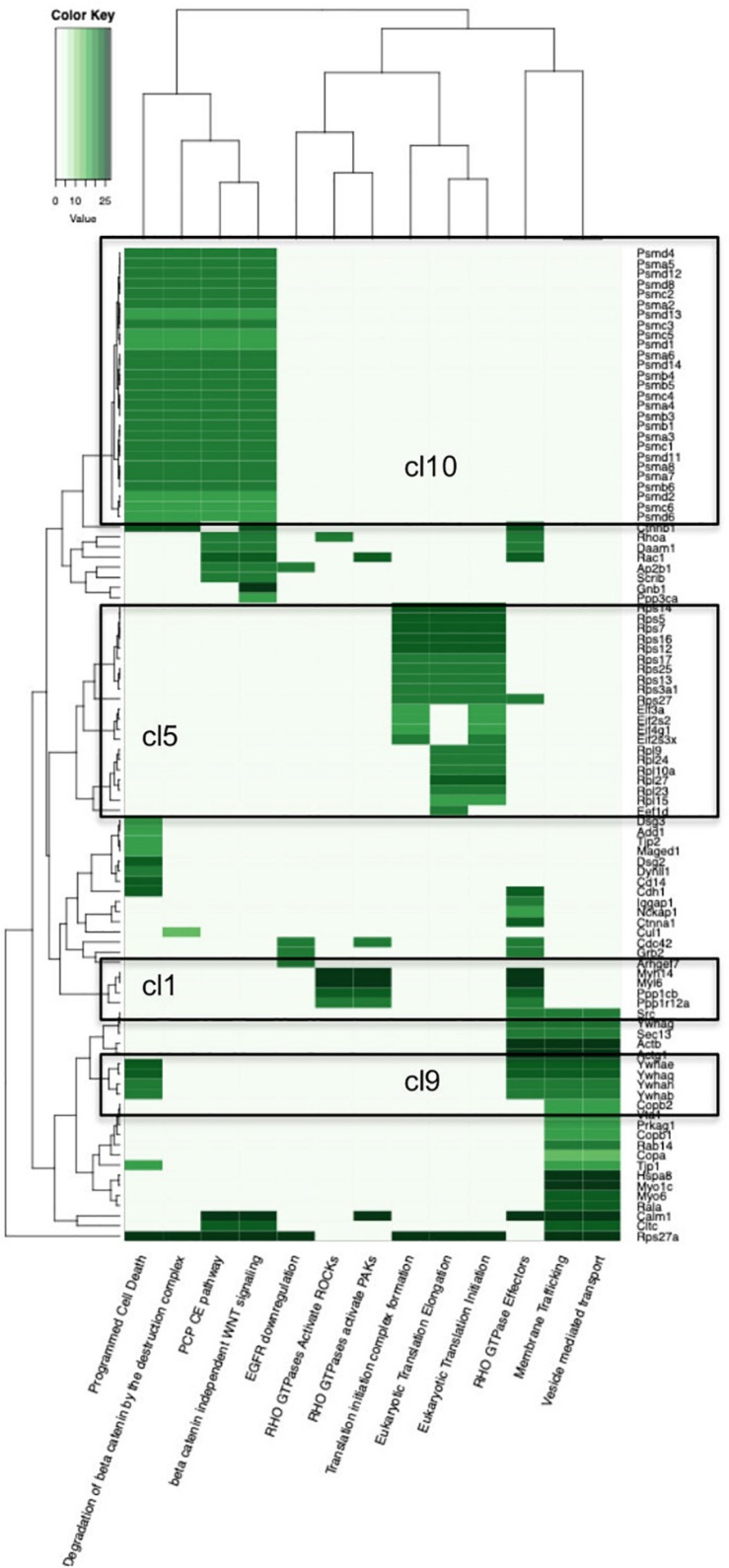
Heatmap of the Reactome Pathway enrichment analysis clustering. Color intensity is based on the average relative protein abundance in MS. Blocks with numbers correspond to respective cluster on the PPI network where this group of proteins belongs.

To obtain a functional view of the p140Cap cancer interactome, we tested its enrichment against Gene Ontology (GO), KEGG and Reactome databases (Supplementary Table S2). According to GO Ontology, the 373 p140Cap-interacting proteins were significantly enriched for a number of GO Cellular Compartment (CC) terms. In particular the enrichment in “Cell- substrate junction” (*P* = 4.96E-39) and “Focal adhesion” (*P* = 8.47E-39) terms (were P - *p*-value, adjusted by multiple testing) indicates that p140Cap protein complexes mediates cell communication, which is consistent with the previously described role for p140Cap in cell-matrix and cell-cell adhesion (Di Stefano et al., 2007; Damiano et al., 2010). In addition, we found that the high significance in “Proteasome complex” (*P* = 1.32E-27), “Endopeptidase complex” (*P* = 1.32E-27) and “Extrinsic component of plasma membrane” (*P* = 4.73E-12) terms, indicates a previously unknown role for p140Cap complexes in protein homeostasis in breast cancer. In the enrichment analysis of GO Biological Process (BP) terms, the most significantly enriched terms included “Regulation of mRNA stability” (*P* = 1.5E-23), “Response to tumor necrosis factor”(*P* = 6.93E-14) and other terms related to regulation of protein translation, DNA and RNA damage response, apoptosis and cell-cycle. Particularly significant is the “Wnt signaling pathway, planar cell polarity pathway” term (*P* = 2.95E-33), indicating that the p140Cap interactome may take part in the Wnt mechanism, a fundamental regulator of cell proliferation in cancer cells (Basu et al., 2018). This is in agreement with the Reactome pathway database, which revealed the overrepresentation for Planar Cell Polarity “PCE/CE pathway” (*P* = 1.04E-30), while “AUF1 (hnRNP D0) destabilizes mRNA” (*P* = 2.47E-31) term highlights a functional role in RNA degradation. “Regulation of Apoptosis” (*P* = 1.29E-30), “Stabilization of p53” (*P* = 4.05E-30), “Ubiquitin –dependent degradation of Cyclin D1” (*P* = 1.6E-32) are also found highly enriched. Top enrichment terms are shown in Table 1 and Figure 2, while the full lists can be found in Supplementary Table S2. As shown in the GO CC terms, also in the Reactome, the terms Cell-Cell communication (*P* = 6.04E-05) and Cell-cell junction organization (*P* = 0.00047817) are still highly significative. Taken together, the functional enrichment analysis from these two distinct sources (GO and Reactome) indicate that the p140Cap interactors exhibit functions relevant to cell adhesion, protein homeostasis, regulation of basic cell features such as cell cycle and apoptosis, which are commonly deregulated in tumor cells.

**TABLE 1:** Top enrichment terms for cancer P140Cap interactome.

**Annotation**	**Annotation terms**	***P*.adjust**
**type**		
GO CC	Cell-substrate junction	4.96E-39
	Focal adhesion	8.47E-39
	Proteosome complex	1.32E-27
	Endopeptidase complex	1.32E-27
	Extrinsic component of plasma membrane	1.20E-12
GO BP	Wnt signaling pathway, planar cell polarity pathway	2.95E-33
	Positive regulation of ubiquitin-protein ligase activity	2.83E-30
	involved in regulation of mitotic cell cycle transition	
	Regulation of mRNA stability	1.50E-23
	TNF- regulated signaling pathway	4.21E-19
	Positive regulation of cellular catabolic process	3.05E-16
GO MF	Cadherin binding involved in cell-cell adhesion	2.31E-25
	Threonine-type endopeptidase activity	3.16E-13
	GTP binding	1.02E-10
Reactome	Vif-mediated degradation of APOBEC3G	8.20E-32
	Regulation of activated PAK-2p34 by proteasome	1.14E-30
	mediated degradation	
	Regulation of apoptosis	1.29E-30
	Ubiquitin-dependent degradation of Cyclin D1	1.29E-30
	Stabilization of p53	4.05E-30
	G1/S DNA damage checkpoints	2.09E-28

### Community Structure Reveals the Topology-Functional Relationships Within p140Cap Interactome

To examine whether the identified functional terms are associated with specific sub complexes within the interactome, we reconstructed the PPI network to perform enrichment analysis over its community structure.

**FIGURE 3:**
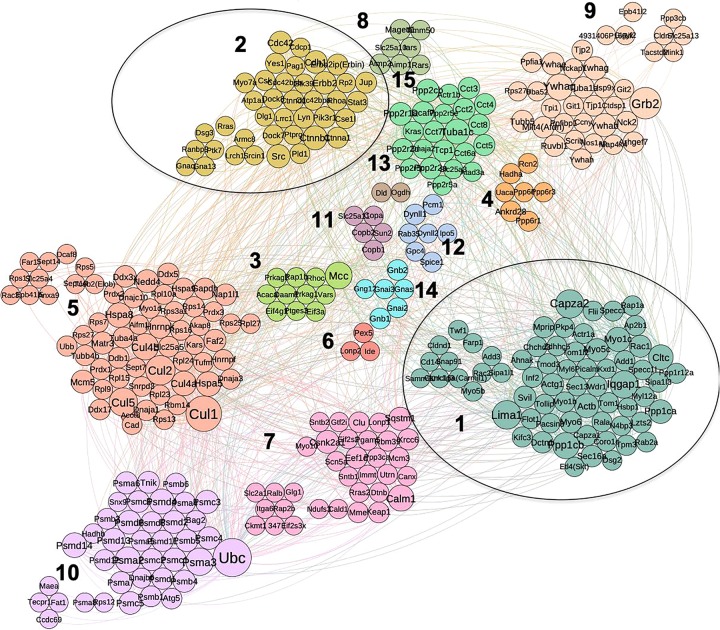
Community structure of p140Cap protein complex in breast cancer obtained by spin-glass algorithm. Fifteen distinct communities are highlighted in different colors; clusters that overlap with synaptic network (1 and 2) are circled.

Using combined mouse and human PPI data collected from three data sources (see section “Materials and Methods”) we built a PPI network for the 374 proteins obtained for the cancer interactome. The network analysis is solely based on our set of PPIs filtered from the Intact, BioGrid and DIP databases: these PPIs are the “direct and physical” human PPIs found from experiments. The resulting network was analyzed with respect to node centrality measures (Supplementary Table S3) including: Degree, Betweenness (Bet), Closeness, Clustering Coefficient (CC), Page Rank (PR), Semi-Local centrality (SL), and mean shortest path (SP). For the remaining analysis, we took the Largest Connected Component (LCC) of the PPI proteome network: 348 nodes and 1630 edges. The LCC was clustered using several algorithms (see “Materials and Methods”, Supplementary Table S3). Hereafter, we show the results of the SpinGlass (sgG1) algorithm, which gives a reasonably small number of communities (Heine et al., 2015), as detailed in Figure 3. For each community structure, degree of nodes is reflected by their diameter.

We performed enrichment analysis for the independent communities with respect to the protein function, protein domain and disease enrichment. For clarity, we named the communities/cluster of cancer network from C1 to C15. We found distinct network communities significantly associated with specific functions (Figure 3 and Supplementary Table S3, where it is possible to sort out the genes belonging to each cluster looking at the column sgG1). For example, the Cluster 2, C2 (comprising 39 proteins) contains key known molecules for p140Cap signaling, including p140Cap itself (SRCIN1), Src, ERBB2 and ERBB2IP (ERBIN), and is highly enriched with kinase domain containing proteins (*P* = 1.5E-06), which suggests that these components of the p140Cap interactome plays a major role in signaling cascades. This cluster is also associated with the most of the tested cancer-related terms, e.g., “Breast cancer” (*P* = 2.2E-04), “Melanoma” (*P* = 5.82E-05), “Colon cancer” (*P* = 1.84E-03), “Stomach carcinoma” (*P* = 9.07E-03), “Malignant glioma” (*P* = 2.1E-04), and with one of synaptopathologies -“Autism spectral disorder” (*P* = 2.28E-03). From a functional perspective, C2 is enriched in the following GO BP terms: Cell junction assembly (*P* = 4.2E-04), “Adherens junction organization” (7.27E-06), ‘Fc gamma receptor signaling pathway” (*P* = 6.04E-03), “Ephrin receptor signaling pathway” (*P* = 1.77E-02) and “Actin filament bundle assembly” (*P* = 2.5E-02) (Supplementary Table S4). Notably, C2 contains 7 proteins shared with synaptic dataset, namely: p140Cap, ERBB2IP, and the cell adhesion proteins such as the Junction plakoglobin (JUP), a common junctional plaque protein, together with the Catenin beta-1 and Catenin delta-1. In addition C2 also contains two proteins involved in intracellular membrane trafficking, the GTPase-activating protein (GAP) RP2 involved in trafficking between the Golgi and the ciliary membrane, and the RANBP9, a protein associated with the small GTP binding protein RAN, which is essential for the translocation of RNA and proteins through the nuclear pore complex.

Cluster 1 (C1) contains another 14 proteins, shared with synaptic interactome, among them, Actinin B, FLII, and SIPA1L1, all involved in actin cytoskeleton remodeling, several members of the F-actin capping protein (CAPZA1, CAPZA2, ADD1, ADD3), and Myosin 6 (MYO6) as a reverse-direction motor protein that moves toward the minus-end of actin filaments, PKP4 (which plays a role as a regulator of Rho activity during cytokinesis and may play a role in junctional plaques), TOM1L2 with a role in protein transport. We also detected in C1 the Skt protein, which belongs to the same family as p140Cap, and Flotillin, a protein that localizes to the caveolae, and plays a role in vesicle trafficking. Similar to C2, we find that C1 is overrepresented with “Ephrin receptor signaling pathway” (*P* = 1.17E-02) and “Actin filament bundle capping” (*P* = 2.2E-04) (Supplementary Table S4).

Other communities also aggregate functionally related proteins together. For example, cluster 10 (C10) contains 39 proteins, among which the proteasome and ubiquitin -related proteins dominate. Moreover, C10 is enriched with the majority of the Reactome terms that were found enriched in entire p140Cap interactome, e.g., “Regulation of Apoptosis”, “Regulation of mitotic cell cycle”, “Signaling by Wnt”, and other terms related to DNA damage response, protein polyubiquitination, EGF, TNF, and NF-KappaB signaling pathways.

Cluster 5 (C5) contains 60 proteins, the majority of which are ribosomal and is associated with “Eukaryotic translation”- related terms, while C15 (21 proteins) is enriched in chaperone-related proteins (*P* = 6.54E-11) and associated with Protein folding (*P* = 4.49E-09). Cluster 7 (C7) (34 proteins), is associated with “Small GTPase Ras signaling” (*P* = 3.4E-02) and “Nuclear transcribed mRNA catabolic process” (*P* = 3.65E-08) terms and, simultaneously, with both cancer and neurological diseases, e.g., “Breast cancer” (*P* = 7.9E-04), “Focal epilepsy” (*P* = 9.29E-03) and “Stomach carcinoma” (*P* = 2.23E-02); likewise C9 is enriched with “Bipolar disorder” (*P* = 4.3E-03), “Schizophrenia” (*P* = 3.89E-03) and “Central nervous system cancer” (*P* = 1.75E-02) terms.

**FIGURE 4:**
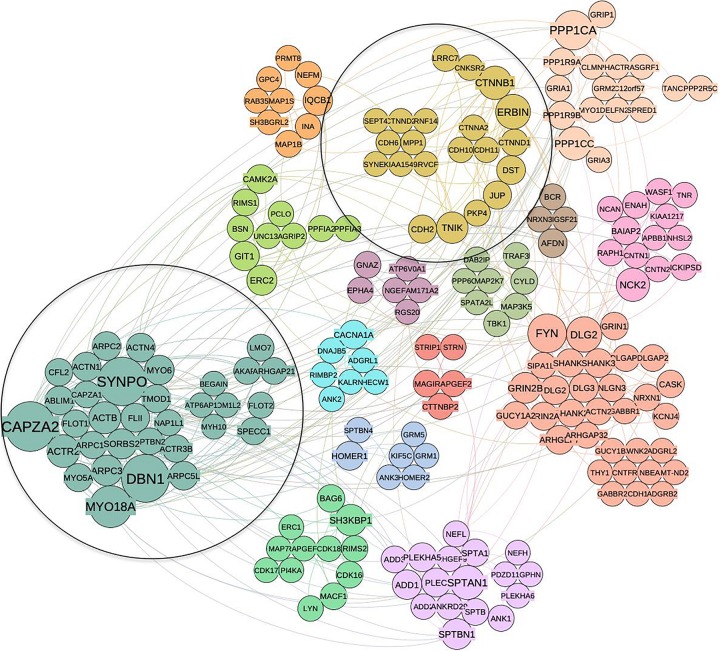
The community structure of p140Cap network in the synaptic compartment and the overlap with cancer set. Similar as for cancer network, distinct communities are highlighted by different colors. Two communities overlapping with cancer network are circled.

Using the p140Cap synaptic interaction from Alfieri et al. (2017), we constructed the corresponding neuronal PPI network (351 interacting proteins with a LCC of 201 nodes and 458 edges). This network was split to communities the same way as above, resulting in 15 clusters (Figure 4). As expected, in the neuronal network we found clusters associated with synaptic transmission and assembly (Neuronal Cluster N1, 32 proteins), and neurotransmitter secretion (N2, four proteins) (Supplementary Table S4).

When compared to cancer interactome, two communities were found to overlap significantly: C2 in cancer network corresponds to N13 in the synaptic one (*P* = 0.03) while C1 corresponds to N4 (*P* = 0.05). N13 contains proteins common to C2, such as Cadherin 6, Catenin alpha-2, Catenin beta-1, Catenin delta-1, JUP and Erbb2IP associated with cell junction assembly (*P* = 3.44E-05) and adherens junction organization (1.06E-04) functions. Similar to C2, we find the N13 cluster is enriched with cancer-related terms, such as SCC, Malignant Glioma (MG) and Hepatocellular Carcinoma.

Thus, the mixed enrichment for cancer and neural diseases terms over the similar clusters likely indicates shared molecular mechanisms for both types of diseases based on common signaling pathways.

### Influential Network Components Are Associated With Disease Terms

To investigate influential nodes in our clustered PPIs from both interactomes we estimated the topological property Semi-local centrality *Clv* (Chen et al., 2010) and the clustering measure Bridgeness *Bv* (Nepusz et al., 2008) as described in Methods. We considered genes influential when found to be topologically important i.e., when they affect or influence other clusters than those they belong to (see section “Materials and Methods” for details). This property enables them with potential to participate in several communities simultaneously and, thus, facilitate spreading signals, which is especially important for disease mechanisms.

**FIGURE 5:**
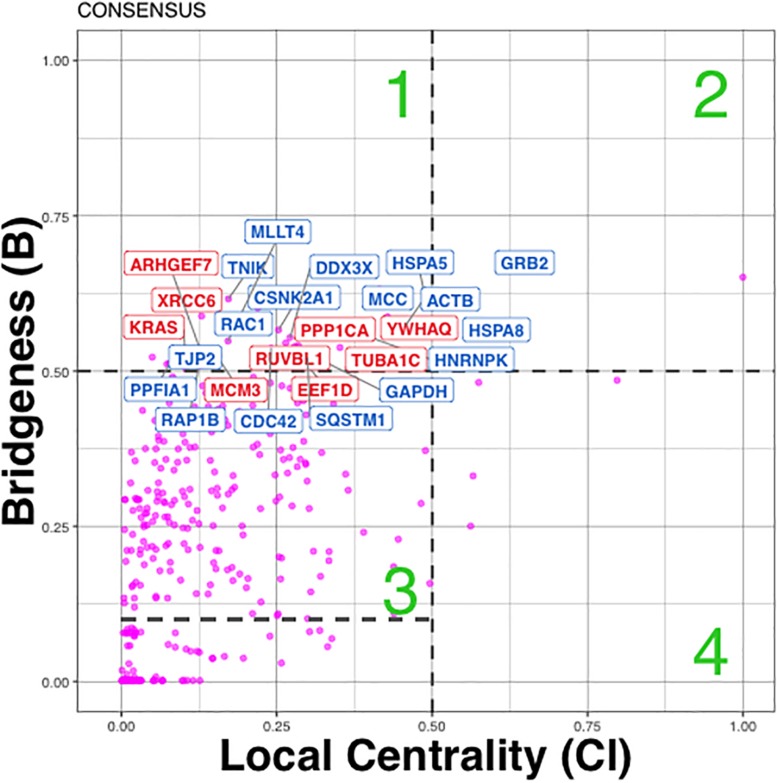
Distribution of influential/bridging proteins in cancer p140Cap networks estimated from consensus clustering results. Proteins were divided into four quadrants or regions labeled 1–4: (1) Bridging proteins with ‘global’ rather than ‘local’ influence (also been called bottle-neck bridges. (2) Bridging proteins with mixed “global” and “local” influence in the network. (3) Proteins important primarily within one or two communities. (4) Proteins that influence just “locally” in the network. Red color corresponds to Bridging proteins annotation with cancer- related diseases only, blue – to proteins annotated with both cancer and neurological diseases.

In total in cancer network we found 38 (36 + 2) Bridging proteins, confidently grouping in Regions 1 and 2 (Figure 5). Region 1 includes bridging proteins with “global” rather than “local” influence, while Region 2 - bridging proteins with mixed “global” and “local” influence in the network (e.g., GRB2), which means they influence the network and its clusters both locally and globally (Figure 5).

The candidate bridging protein subset was analyzed with respect to function and disease annotation. Proteins from Region 1 were significantly enriched with Breast Cancer (23/41, *P* = 2.07E-04) and Gastro Intestinal System cancer (GIS) (23/41, *P* = 2.7E-03) terms, and Nervous System Cancer (CNS) (9/41, *P* = 0.03) (Figure 5, where cancer terms are highlighted in red). Of those, 17 proteins were associated with both cancer and synaptic terms (Figure 5, highlighted in blue, Supplementary Table S5). Among them, we found: (a) regulators of actin cytoskeleton remodeling and cell motility (Actinin B, RAC1 CDC42, PPFIA1 alias Liprin); (b) cell adhesion proteins (RAP1B involved in junctional adhesion, JTJP2 encoding a zonula occludens member, and MLLT4 also known as Afadin which, probably together with the E-cadherin-catenin system, plays a role in the organization of homotypic, interneuronal and heterotypic cell-cell adherens junctions); (c) modulators of the Wnt/b-catenin pathway (MCC that suppresses cell proliferation and TNIK, a serine/threonine kinase that acts as an essential activator of the Wnt signaling pathway); (d) SQSTM1 also called p62, a multifunctional protein that binds ubiquitin and regulates activation of the nuclear factor kappa-B (NF-kB) signaling; (e) a key growth factor receptor adaptor such as GRB2; (f) the heat shock chaperones HSPA5, HSPA8; (g) antiapoptotic proteins such as DDX3X and CSNK2A1, the catalytic subunit of a constitutively active serine/threonine-protein kinase complex that regulates numerous cellular processes, such as cell cycle progression, apoptosis and transcription. Additional proteins like GAPDH, and a major pre-mRNA-binding protein HNRNPK, were also detected as bridging proteins.

Bringing into consideration each Bridging protein’s annotation (Region 1) with GO Biological Process (BP), we identified significant overlap between GIS and CNS cancer terms and following functional terms (see section “Materials and Methods”): small GTPase mediated signal transduction (*P* = 5.5E-4, 3.7E-4), Ras protein signal transduction (GO:0007265 *P* = 2.2E-03, 2.0E-04), innate immune response (GO:004087 *P* = 1.0E-04, 2.6E-02), axon guidance (GO:0007411 *P* = 1.9E-03, 2.6E-02) and neurotrophin TRK receptor signaling pathway (GO:0048011 *P* = 1.7E-02, 2.5E-03) (Supplementary Table S5). This overlap might indicate the pathways involved in disease.

### Disease Modules Overlap on the p140Cap Interactomes

A simplistic view of the two interactomes would suggest that the disease enrichments for neurological or cancer terms would segregate into the tissue specific regions of the network. Conversely the neurological disease and cancer related terms overlapping with each other on the PPI networks, would suggest common signaling pathways impacting on shared biology. We tested the gene-disease annotation (GDA) data on p140Cap synaptic and Cancer PPI networks, using a network based approach to identify the location of disease modules, localized regions of connections between disease-related proteins, on the interactome (Menche et al., 2015). We tested the overlaps between GDA data on PPI network with independent method based on network topology (see “Materials and Methods” for details). Here, by definition, “Disease modules” are localized regions of connections between disease-related proteins in interactome (Menche et al., 2015). The full list of diseases and abbreviations is shown in Table 3.

Within the cancer terms we found significant overlap between Breast Cancer and Gastrointestinal System Cancer (*P* = 4.03E-6), Breast Cancer and Stomach Cancer (1.56E-4), Breast Cancer and Malignant Glioma (*P* = 2.42E-5), Melanoma and SCC (1.41E-5), Melanoma and Malignant Glioma (*P* = 1.92E-5) (see the full list of disease-disease pairs in Supplementary Table S6).

Similarly to what we found on Bridging proteins and communities level, the neurodegenerative diseases, such as Alzheimer’s disease (AD), Epilepsy (Epi), Parkinson disease (PD), Huntington disease (HD) and Frontotemporal Dementia (FTD) show repeated evidence of overlapping with cancer related terms. This includes not only CNS cancer terms, such as Neuroblastoma (NB), Autonomus Nervous System Neoplasm (ANSN), Peripheral Nervous System Neoplasm (PNS), Neural System Cancer (NSC), Central Neural System Cancer (CNSC), Malignant Glioma (MG) but also Gastrointestinal cancer terms: Stomach Cancer (SC), Gastrointestinal Cancer (GISC), along with Breast Cancer (BC), Melanoma (MEL) and Squamous Cell Carcinoma (SCC).

We found these overlaps most significant for Alzheimer’s Disease with Neural System Cancer (*P* = 1.9 × 10-4), Peripheral Nervous System Neoplasm (P 5.8 × 10-3), Autonomus Nervous System Neoplasm (*P* = 8.8E-03), NB (*P* = 0.01) and Malignant Glioma (*P* = 0.02). AD is also found to overlap significantly with SCC (*P* = 0.02), BC (*P* = 5.2E-4) and Melanoma (*P* = 1.0E-3). Of the other neurological disease, Schizophrenia annotations overlap with Stomach Cancer (1.92E-5) and Parkinson disease overlaps significantly with SCC (*P* = 0.02) (Supplementary Table S6).

### Comparison of Cancer and Synaptic p140Cap Interactomes

**FIGURE 6:**
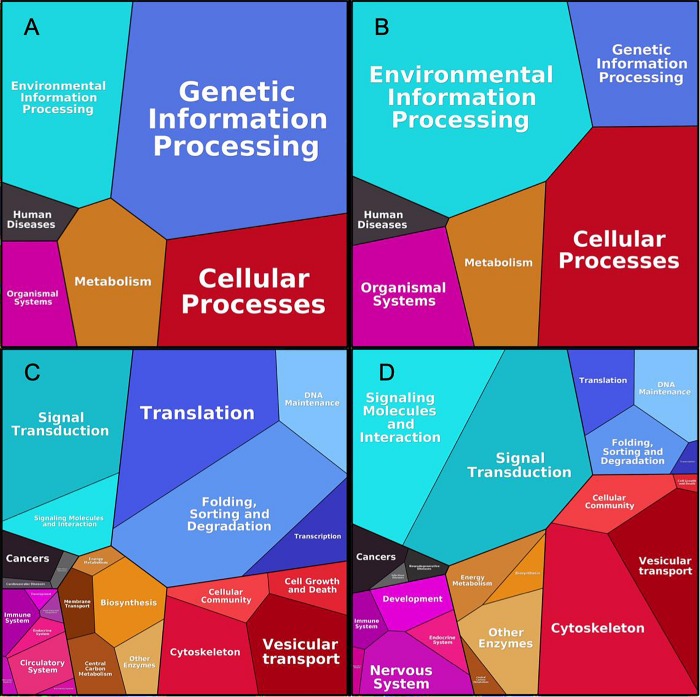
Proteomaps for cancer and synaptic sets. As shown in Liebermeister et al. (2014), upper figures correspond to high-level KEGG functional division for p140Cap Cancer **(A)** and Synaptic **(B)** proteome; Bottom figures correspond to respective lower hierarchy terms for Cancer **(C)** and Synaptic **(D)** proteomes.

Figure 6 shows the generalized functional profiles for both interactomes based on the proteins investment into primary biological function, such as Processing of Genetic Information, Metabolism, Signaling, Transport, etc., which in turn are subdivided to lower level KEGG categories (Liebermeister et al., 2014). Here, size of category/pathway depends on accumulated abundances of proteins, participating in the respective pathways. The categories are equivalent for both interactomes, however, the dominant terms are evidently different. While “Genetic Information Processing” (mainly with “Translation” and “Folding, Sorting and Degradation” terms) makes the largest impact in cancer interactome (Figures 6A,C), in the synaptic dataset the most prominent category is “Environmental Information Processing”, equally distributed between “Signaling molecules and interactions” and “Signal transduction terms” (Figures 6B,D). Metabolism category in general is equally represented in both interactomes, but specific terms, such as “Biosynthesis” and “Central Carbon Metabolism” are more represented in cancer. In the synaptic interaction, Cellular Processes like “Cytoskeleton” and “Vesicular transport” are more prevalent.

This is also reflected in the GO and Reactome pathway enrichment analysis performed on both protein lists. Most of enriched terms appear to be context–specific, however, a few of them are shared between the lists. In particular, for GO CC (Cellular Compartment) the most enriched terms for both networks are “Actin cytoskeleton”(*P* = 1.62E-28), “Cell-substrate junction” (*P* = 4.96E-39), “Cell-cell adherens junction” (*P* = 2.5E-26) (see Table 2 for comparison Supplementary Table S2 for the full list of enriched terms). Similarly, the most enriched BP (Biological Process) terms for both networks is “Actin filament organization” (*P* = 2.06E-18), while the common Reactome and KEGG pathways include Rho GTPase signaling and cell-cell adherens and junction (Table 2).

**TABLE 2:** Top enrichment terms specific either for synaptic p140Cap interactome (column 1) or for cancer p140Cap interactome (column 2), and common for both (column 3).

**Annotation**	**Synaptic p140Cap interactome**	**Cancer p140Cap interactome**	**Overlap**
GO CC	Excitatory synapse	Proteosome complex	Actin cytoskeleton
	Postsynaptic density	Endopeptidase complex	Cell-substrate junction
	Ionotropic glutamate receptor complex	Extrinsic component of plasma membrane	Cell-cell adherens junction
GO BP	Modulation of synaptic transmission	Wnt signaling pathwayPlanar cell polarity pathway	Neurotrophin TRK receptor signaling pathway
			Innate immune response
			Actin filament organization
	Synapse organization	Regulation of mRNA stability	Axon guidance
	Neurotransmitter transport	Response to TNF	Actin binding
GO MF	Receptor signaling complex scaffold activity	Heat shock protein binding	GTPase regulator activity
	PDZ domain binding	Protein binding involved in cell-cell adhesion	Calmodulin binding
	Scaffold protein binding	G-protein beta/gamma-subunit complex binding	Tight junction
KEGG	Endocytosis	Proteasome	Regulation of actin cytoskeleton
	Glutamatergic synapse	Pathways in cancer	Adherens junction
	Long-term potentiation	Ribosome	Ras signaling pathway
			Signaling by Rho GTPases
Reactome	Trafficking of AMPA receptors	p53-Independent DNA Damage Response	RHO GTPases activate CIT
	Unblocking of NMDA receptor, glutamate binding and activation	AUF1 (hnRNP D0) destabilizes mRNA	Cell-Cell communication
	Interaction between L1 and ankyrins	Ubiquitin-dependent degradation of Cyclin D1	Cell junction organization
	Ras activation uopn Ca2 + influx through NMDA receptor	Regulation of Apoptosis	Adherens junctions interactions
	GABA receptor activation	Regulation of DNA replication	

Direct comparison of two lists of proteins found in cancer model cells (here) and in neurons (Alfieri et al., 2017; Supplementary Table S1) identified 39 genes in common (*P* = E-22) (Supplementary Table S1), which correspond to “shared” interactome. Within the network models, the majority of these proteins are concentrated in clusters C1 and C2 of breast cancer network, and N4 and N13 of synaptic network, respectively. As would be expected, they are associated with GO terms common for both interactomes (Table 3). Pathway enrichment analysis performed on the 39 overlapping genes confirmed that all the terms listed above are significantly enriched (Table 4).

**TABLE 3:** Disease enrichment values for p140Cap cancer and synaptic PPI networks.

	**Cancer**				**Synaptic**				
	**PPI network**				**PPI network**				
**Disease**	**HDO ID**	**N**	***P*-value**	**BC**	**B-Y**	**N**	***P*-value**	**BC**	**B-Y**
Schizophrenia (SCH)	DOID:5419	55	1.5E-4		0.2	71	6.3E-20	^∗∗∗^	1.9E-15
Alzheimer’s disease (AD)	DOID:10652	47	1.8E-4		0.2	41	5.2E-7	^∗∗^	3.5E-3
Autism spectrum disorder (ASD)	DOID:0060041	11	0.5		1	24	1		1
Autistic disorder (AUT)	DOID:12849	11	0.5		1	24	1.1E-7	^∗∗∗^	1.7E-3
Bipolar disorder (BD)	DOID:3312	18	0.8		1	28	2.6E-4		0.6
Epilepsy syndrome (Epi)	DOID:1826	19	0.3		1	24	0.3		1
Temporal lobe epilepsy (TLE)	DOID:3328	1	0.9		1	11	5.8E-7	^∗∗^	3.5E-3
Focal epilepsy (Fepi)	DOID:2234	2	0.8		1	13	0.1		1
Parkinson’s disease (PD)	DOID:14330	29	5.2E-6	^∗^	0.02	14	0.02		1
Frontotemporal dementia (FTD)	DOID:9255	11	9.6E-4		0.7	10	1.3E-4		0.3
Huntington’s disease (HD)	DOID:12858	5	0.4		1	4	0.3		1
Intellectual disability (ID)	DOID:1059	15	0.3		1	29	2.2E-7	^∗∗∗^	2.2E-3
Neuroblastoma (NB)	DOID:769	14	0.2		1	11	0.1		1
Autonomic nervous system neoplasm (ANSN)	DOID:2621	14	0.2		1	11	0.1		1
Peripheral nervous system neoplasm (PNSN)	DOID:1192	17	0.1		1	12	0.1		1
Nervous system cancer (NSC)	DOID:3093	44	0.2		1	25	0.1		1
Central nervous system cancer (CNSC)	DOID:3620	23	0.4		1	14	0.2		1
Malignant glioma (MG)	DOID:3070	43	0.1		1	23	0.06		1
Stomach cancer (SC)	DOID:10534	22	0.01		1	4	0.9		1
Gastrointestinal system cancer (GISC)	DOID:3119	129	0.2		1	58	0.2		1
Stomach carcinoma (SCA)	DOID:5517	18	0.06		1	3	0.9		1
Gastric adenocarcinoma (GAC)	DOID:3717	7	0.6		1	1	0.9		1
Gastric lymphoma (GLC)	DOID:10540	1	0.4		1	−	−		−
Breast cancer (BC)	DOID:1612	108	2.8E-7	^∗∗∗^	2.8E-3	43	0.4		1
Melanoma (MEL)	DOID:1909	52	1.3E-8	^∗∗∗^	3.9E-4	23	0.03		1
Hepatocellular carcinoma (HCC)	DOID:684	69	3.2E-8	^∗∗∗^	4.8E-4	28	0.2		1
Squamous cell carcinoma (SCC)	DOID:1749	54	1.1E-6	^∗∗^	8.3E-3	22	0.2		1

*Enrichment values for our set of common synaptopathies and cancers for p140Cap PPI proteome networks using combined OMIM/geneRIF/Ensembl variation annotation data mapped into the full Human Disease Ontology (HDO) ontology tree (17,731 genes annotated onto 3457 HDO terms) for the cancer (321 significant genes annotated onto 1346 HDO terms), synaptic (212 significant genes annotated onto 955 HDO terms) networks. Where enrichment values were calculated using the Topology-based Elimination Fisher method (Alexa et al., 2006), and N gives the number of disease genes found in each network. Multiple hypothesis corrections using the Bonferroni test (BC), at the 0.001 (^∗∗∗^), 0.01 (^∗∗^) and 0.05 (^∗^) significance levels, and the Benjamini and Yekutieli (B-Y) (Benjamini and Yekutieli, 2001)procedure is shown for each network.*

**TABLE 4:** Functional enrichment for 39 shared proteins.

**Term ID**	**Ontology**	**Description**	***p*-value**	***p*.adjust**
GO:0034330	GO BP	Cell junction organization	3.22E-09	4.15E-06
GO:0048013	GO BP	Ephrin receptor signaling pathway	4.09E-08	2.64E-05
GO:0034332	GO BP	Adherens junction organization	2.54E-07	1.09E-04
GO:0034329	GO BP	Cell junction assembly	4.38E-06	1.05E-03
GO:0030837	GO BP	Negative regulation of actin filament polymerization	5.74E-06	1.05E-03
GO:0051016	GO BP	Barbed-end actin filament capping	8.71E-06	1.4E-03
GO:0034113	GO BP	Heterotypic cell-cell adhesion	1.55E-04	9.08E-03
GO:0005913	GO CC	Cell-cell adherens junction	1.38E-07	8.27E-08
GO:0005925	GO CC	Focal adhesion	3.84E-07	2.30E-07
GO:0005924	GO CC	Cell-substrate adherens junction	3.84E-07	2.30E-07
GO:0015629	GO CC	Actin cytoskeleton	1.06E-06	6.34E-07
GO:0045121	GO CC	Membrane raft	2.68E-03	1.6E-03
GO:0050839	GO MF	Cell adhesion molecule binding	5.95E-08	7.32E-06
GO:0003779	GO MF	Actin binding	2.06E-07	7.79E-06
hsa04520	KEGG	Adherens junction	5.09E-05	2.06E-03
hsa04611	KEGG	Rap1 signaling pathway	5.16E-03	4.29E-03
hsa04810	KEGG	Regulation of actin cytoskeleton	3.18E-03	5.16E-03
hsa04530	KEGG	Tight junction	1.37E-03	1.59E-02
hsa04144	KEGG	Endocytosis	5.11E-03	4.8E-02
2682334	REACTOME	EPH-Ephrin signaling	1.45E-07	1.00E-05
418990	REACTOME	Adherens junctions interactions	2.82E-08	3.89E-06
421270	REACTOME	Cell-cell junction organization	3.39E-07	1.56E-05
4420097	REACTOME	VEGFA-VEGFR2 Pathway	1.62E-04	3.2E-04
1266738	REACTOME	Developmental Biology	2.42E-04	3.34E-03
5626467	REACTOME	RHO GTPases activate IQGAPs	9.45E-04	1.0E-02

Thus, the functional signature of two interactomes is determined primarily by its context: organ/tissue and condition specificity; while the overlap provides a list of shared functional terms, which are likely to be associated with p140Cap’s core molecular function.

## Discussion

Due to the role of p140Cap as a scaffold protein, and to the results obtained analyzing the interactome in the synaptic compartment (Alfieri et al., 2017), we reasonably assumed that in breast cancer cells p140Cap would also bind to a large number of intracellular proteins, influencing breast cancer biology. Proteomic analysis of p140Cap interactome in the ERBB2 breast cancer model uncovered the 373 interacting proteins described here. Amongst these, we found an enrichment in several Gene Ontology terms involved in cell-substrate junction, focal adhesion organization and cell-cell adhesion and in Reactome terms (i.e., Regulation of apoptosis), including functions relevant to cell adhesion, protein homeostasis, regulation of cell cycle and apoptosis. In other words, the complex was enriched for molecules whose functions are associated with those frequently deregulated in cancer.

In the ERBB2 cell model chosen for this analysis, p140Cap is causal in impairing *in vivo* tumor growth and metastasis formation. This model as well the double transgenic mice p140-NeuT are consistent with the overall improved prognosis observed in the human ERBB2-positive breast cancer cohort (Grasso et al., 2017). Included in those proteins found to interact with p140Cap, E-cadherin and the Catenin beta-1, have been already found associate to p140Cap by co-immunoprecipitation experiments in MCF-7 cells, a typical luminal A breast cancer model (Damiano et al., 2010). Taken together, these data indicate that these two interacting proteins can associate to p140Cap in at least two different breast cancer subtypes (ERBB2-positive versus Luminal A subtypes). Therefore, we can assume that the proteins identified in the breast cancer interactome, are “bona fide” interactors, and that the p140Cap-dependent interaction may affect their biological functions.

We already know that in MCF7 cells, the presence of p140Cap exerts a critical role in E-cadherin stabilization at the cell membrane (Cabodi et al., 2010), while in the ERBB2 model here described p140Cap expression determines an increased expression of E-Cadherin at the cell surface in *in vivo* tumors. The increase in E-Cadherin expression at the cell membrane is accompanied by a reversion of the so-called “cadherin switch” (that is, increase of the mesenchymal marker N-cadherin and a concomitant decrease of the epithelial marker E-cadherin), which is a canonical hallmark of EMT in cancer (Hanahan and Weinberg, 2011; Lamouille et al., 2014; Bill and Christofori, 2015), further confirmed by the concomitant decrease in EMT markers (Grasso et al., 2017). Moreover, to further demonstrate that the binding with p140Cap may affect the function of specific interactors, it has been recently shown that Catenin beta1 regulates presynaptic function through its direct binding to p140Cap (Li et al., 2017). Overall, from these data we could suggest that through binding to p140Cap, these interactors may modulate their proper function in the tumors.

The putative role of p140Cap in proteasome complex, regulation of mRNA stability and DNA damage checkpoint opens new perspectives on functional different roles of p140Cap in breast cancer cells. Indeed, we recently provided the first evidence that the SRCIN1/p140Cap adaptor protein is a key player in neuroblastoma as a new independent prognostic marker for patient outcome and treatment (Grasso et al., 2019). In neuroblastoma cells p140Cap increases cell sensitivity to chemotherapy-induced DNA damage (Grasso et al., 2019), suggesting that p140Cap could interact with proteins involved in DNA damage sensitivity in breast cancer cells.

Community analysis based on network topology suggests that the p140Cap interactome comprises 15 functionally independent clusters. This subdivision into clusters allows us to identify subsets of proteins that preferentially contribute to specific functions. For example, Cluster C2 contains p140Cap and the tyrosine kinases Src and Erbb2, reinforcing the concept that p140Cap can associate and regulated tyrosine kinases (Di Stefano et al., 2007; Bagnato et al., 2017), which play key roles in breast cancer transformation and progression.

Our study provides a first look at similarities and differences between the p140Cap protein’s interactomes in healthy specialized tissue (brain synaptosome) compared to an aggressive ERBB2 breast cancer model. Comparing across studies is notoriously difficult but the following features gave us confidence: the two p140Cap interactome were both from murine tissue/cells, generated with the same reagents and procedures. We compared both interactomes with the same set of bioinformatics methods including GO enrichment and protein – protein interaction (PPI) network analysis and found distinct signatures for both of them. While the interactome obtained in breast cancer is clearly enriched with terms related to Genetic Information processing, including pathways related to cell cycle, apoptosis, DNA damage, transcription and translation, the proteome obtained in brain is enriched with environmental information processing, including signal transduction and information flow through the synapse. We found that the majority of interacting proteins are clearly different between these two conditions, which likely reflects their tissue, organ and cell specificity. In other words, both proteomes reflect their underlying biological context more obviously than they do each other, despite the common bait protein used to isolate them.

However, 39 proteins are common. When compared to the total mouse genome 24,402 (genes with protein sequence data, taken from MGI website) the probability of observing an overlap of this size from two independent datasets is very low (*P* = 2.68E-22). However, one may argue that datasets are not independent as they have common bait – p140Cap. If we try to estimate the probability of cancer set given we know the neural one:

*P (cancer set | neuronal set)* = *P (cancer set AND neuronal set)/P (neuronal set)*,

we end up with *P* = 5.63E-10, which is again, very significant.

Overall, comparing the list of cancer interactors of p140Cap with the full list of proteins identified in synapse, there are about 160 interacting proteins in cancer that were not identified in synapse, indicating that the cancer interactome could be cell-type specific. On the other hand, other 200 interacting proteins in cancer are also expressed in the synaptic compartment, but do not interact with p140Cap in the synapse. Thus, we can hypothesize that some tissue-specific proteins could be the key regulators of p140Cap interactome.

Despite the relatively small number of common genes, several selected pathways are shared between the two proteomes, e.g., “Actin cytoskeleton”, “Cell-substrate junction”, “Cell-cell adherens junction”, “Fc receptor signaling pathway”, “Actin filament organization” and “Rho GTPase signaling”, “Neurotrophin TRK receptor signaling pathway”.

Comparison of main network properties and community structure of two networks along with their relationship with cellular functions, signaling pathways and diseases revealed, as would be expected given the differing biology, that both networks have distinct community structures associated with condition-specific functions. However, we found common pathways assigned to two specific communities in cancer and synaptic network, which contain the majority of the 39 common proteins.

Notably, the shared pathways listed above feature enrichment for both neurodegenerative diseases and cancer. Some function- disease pairs persist in both interactomes, e.g., “Cell junction assembly” and “Adherens junction assembly” usually co-occur in the same communities alongside cancer –related terms. This may indicate that despite their diversity there are common signaling molecular mechanisms underpinning the function of both interactomes. Similar trends in function-disease overlap were observed for identified Bridging proteins that are likely providing the core signaling framework for p140Cap interactome, and disease-disease relationships studied over PPI network.

Overall, through a bioinformatics approach, these results provide the first interactome profile of p140Cap and the underlined pathways in breast cancer cells, paving the way to experimentally address their role in the tumor suppressing properties of p140Cap in breast cancer.

## Data Availability Statement

The datasets generated for this study can be found in the ProteomeXchange with the dataset identifier PXD008778.

## Author Contributions

JC, OS, and CM designed the research. JC and AAl performed all the biochemistry experiments and analyzed the data. OS, CM, UA, and JA performed all the bioinformatics analysis. AAd and YC performed the mass spectrometry analysis. VS characterized the breast cancer cell model. CA, AM, EM, and MM contributed to the study of the synaptic network. OS, UA, MM, ET, JA, and PD wrote the manuscript. All authors reviewed the manuscript.

## Conflict of Interest

The authors declare that the research was conducted in the absence of any commercial or financial relationships that could be construed as a potential conflict of interest.

## Supplementary Material

FIGURE S1 *In vitro* characterization of p140Cap-TuBo cell line.

FIGURE S2 **(A–C)** Panels represent three distinct TUBO cell extracts that were immunoprecipitated using p140Cap monoclonal antibody. **A**: exp 1, **B**: exp 2; **C**: exp 3. The immunoprecipitates and the corresponding cell extracts (30 micrograms) were run on a 4–15% SDSPAGE and the nitrocellulose membranes were cut according to the molecular weight, in order to decorate the upper part with the p140Cap antibodies and the lower part with the Tubulin antibodies for loading controls. On the left, we show the merge between the colorimetric WB with the chemiluminescent WB, obtained at the ChemiDoc Imaging System from BIO RAD. Membranes were all exposed for 30 sec.

TABLE S1 List of proteins identified and quantified in co-IP eluates from NeuT-TUBO (Mock) and p140-TUBO cells (Oep).

TABLE S2 Enrichment results for cancer and synaptic p140Cap interactomes based on GO (MF, BP, CC), KEGG and Reactome ontologies.

TABLE S3 Community structure for cancer and synaptic p140Cap interactomes. Columns B and C contain the lists of genes and their IDs, columns D-I, R, and S their cluster membership obtained with respective clustering algorithm (spin glass in column I). Columns J to Q contain disease and function annotation terms for each of the genes, T- BG contain the network characteristics of the genes.

TABLE S4 Enrichment results for clusters for cancer and synaptic p140Cap. Spreadsheet 1 contain the specific and overlapping terms for both networks.

TABLE S5 Bridgeness values for cancer and synaptic p140Cap networks.

TABLE S6 Disease overlap for cancer and synaptic p140Cap networks.

## References

[B1] Alexa A. Rahnenfuhrer J. Lengauer T. (2006). Improved scoring of functional groups from gene expression data by decorrelating GO graph structure. *Bioinformatics* 22 1600–1607. 10.1093/bioinformatics/btl140 16606683

[B2] Alfieri A. Sorokina O. Adrait A. Angelini C. Russo I. Morellato A. (2017). Synaptic interactome mining reveals p140Cap as a new hub for PSD proteins involved in psychiatric and neurological disorders. *Front. Mol. Neurosci.* 10:212. 10.3389/fnmol.2017.00212 28713243 PMC5492163

[B3] Aronson A. R. Lang F. M. (2010). An overview of MetaMap: historical perspective and recent advances. *J. Am. Med. Inform. Assoc.* 17 229–236. 10.1136/jamia.2009.002733 20442139 PMC2995713

[B4] Bagnato P. Castagnino A. Cortese K. Bono M. Grasso S. Bellese G. (2017). Cooperative but distinct early co-signaling events originate from ERBB2 and ERBB1 receptors upon trastuzumab treatment in breast cancer cells. *Oncotarget* 8 60109–60122. 10.18632/oncotarget.17686 28947957 PMC5601125

[B5] Basu S. Cheriyamundath S. Ben-Ze’ev A. (2018). Cell-cell adhesion: linking Wnt/beta-catenin signaling with partial EMT and stemness traits in tumorigenesis. *F1000Res.* 7:F1000 Faculty Rev-1488. 10.12688/f1000research.15782.1 30271576 PMC6144947

[B6] Benjamini Y. Yekutieli D. (2001). The control of the false discovery rate in multiple testing under dependency. *Ann. Stat.* 29 1165–1188. 10.1186/1471-2105-9-114 18298808 PMC2375137

[B7] Bill R. Christofori G. (2015). The relevance of EMT in breast cancer metastasis: correlation or causality? *FEBS Let.* 589 1577–1587. 10.1016/j.febslet.2015.05.002 25979173

[B8] Boggio K. Nicoletti G. Di Carlo E. Cavallo F. Landuzzi L. Melani C. (1998). Interleukin 12-mediated prevention of spontaneous mammary adenocarcinomas in two lines of Her-2/neu transgenic mice. *J. Exp. Med.* 188 589–596. 10.1084/jem.188.3.589 9687535 PMC2212479

[B9] Cabodi S. del Pilar Camacho-Leal M. Di Stefano P. Defilippi P. (2010). Integrin signalling adaptors: not only figurants in the cancer story. *Nat. Rev. Cancer* 10 858–870. 10.1038/nrc2967 21102636

[B10] Chatr-Aryamontri A. Breitkreutz B. J. Oughtred R. Boucher L. Heinicke S. Chen D. (2015). The BioGRID interaction database: 2015 update. *Nucleic Acids Res.* 43 D470–D478. 10.1093/nar/gku1204 25428363 PMC4383984

[B11] Chen D. Zhong Q. (2012). A tethering coherent protein in autophagosome maturation. *Autophagy* 8 985–986. 10.4161/auto.20255 22617511 PMC3427267

[B12] Chen Y. Cunningham F. Rios D. McLaren W. M. Smith J. Pritchard B. (2010). Ensembl variation resources. *BMC Genomics* 11:293. 10.1186/1471-2164-11-293 20459805 PMC2894800

[B13] Chin L. S. Nugent R. D. Raynor M. C. Vavalle J. P. Li L. (2000). SNIP, a novel SNAP-25-interacting protein implicated in regulated exocytosis. *J. Biol. Chem.* 275 1191–1200. 10.1074/jbc.275.2.1191 10625663

[B14] Damiano L. Di Stefano P. Camacho Leal M. P. Barba M. Mainiero F. Cabodi S. (2010). p140Cap dual regulation of E-cadherin/EGFR cross-talk and Ras signalling in tumour cell scatter and proliferation. *Oncogene* 29 3677–3690. 10.1038/onc.2010.128 20453886

[B15] Di Stefano P. Cabodi S. Boeri Erba E. Margaria V. Bergatto E. Giuffrida M. G. (2004). P130Cas-associated protein (p140Cap) as a new tyrosine-phosphorylated protein involved in cell spreading. *Mol. Biol. Cell* 15 787–800. 10.1091/mbc.e03-09-0689 14657239 PMC329393

[B16] Di Stefano P. Damiano L. Cabodi S. Aramu S. Tordella L. Praduroux A. (2007). p140Cap protein suppresses tumour cell properties, regulating Csk and Src kinase activity. *EMBO J.* 26 2843–2855. 10.1038/sj.emboj.7601724 17525734 PMC1894765

[B17] Gopalan P. K. Blei D. M. (2013). Efficient discovery of overlapping communities in massive networks. *Proc. Natl. Acad. Sci. U.S.A.* 110 14534–14539. 10.1073/pnas.1221839110 23950224 PMC3767539

[B18] Grasso S. Cangelosi D. Chapelle J. Alzona M. Centonze G. Lamolinara A. (2019). The SRCIN1/p140Cap adaptor protein negatively regulates the aggressiveness of neuroblastoma. *Cell Death Dif.* 10.1038/s41418-019-0386-6 [Epub ahead of print]. 31285546 PMC7205889

[B19] Grasso S. Chapelle J. Salemme V. Aramu S. Russo I. Vitale N. (2017). The scaffold protein p140Cap limits ERBB2-mediated breast cancer progression interfering with Rac GTPase-controlled circuitries. *Nat. Commun.* 8:14797. 10.1038/ncomms14797 28300085 PMC5357316

[B20] Guimera R. Nunes Amaral L. A. (2005). Functional cartography of complex metabolic networks. *Nature* 433 895–900. 10.1038/nature03288 15729348 PMC2175124

[B21] Hanahan D. Weinberg R. A. (2011). Hallmarks of cancer: the next generation. *Cell* 144 646–674. 10.1016/j.cell.2011.02.013 21376230

[B22] Hedman A. C. Smith J. M. Sacks D. B. (2015). The biology of IQGAP proteins: beyond the cytoskeleton. *EMBO Rep.* 16 427–446. 10.15252/embr.201439834 25722290 PMC4388610

[B23] Heine P. Ehrlicher A. Kas J. (2015). Neuronal and metastatic cancer cells: unlike brothers. *Biochim. Biophys. Acta* 1853 3126–3131. 10.1016/j.bbamcr.2015.06.011 26119327

[B24] Jaworski J. Kapitein L. C. Gouveia S. M. Dortland B. R. Wulf P. S. Grigoriev I. (2009). Dynamic microtubules regulate dendritic spine morphology and synaptic plasticity. *Neuron* 61 85–100. 10.1016/j.neuron.2008.11.013 19146815

[B25] Kerrien S. Aranda B. Breuza L. Bridge A. Broackes-Carter F. Chen C. (2012). The IntAct molecular interaction database in 2012. *Nucleic Acids Res.* 40 D841–D846. 10.1093/nar/gkr1088 22121220 PMC3245075

[B26] Lamouille S. Xu J. Derynck R. (2014). Molecular mechanisms of epithelial-mesenchymal transition. *Nat. Rev. Mol. Cell Biol.* 15 178–196. 10.1038/nrm3758 24556840 PMC4240281

[B27] Li M. Y. Miao W. Y. Wu Q. Z. He S. J. Yan G. Yang Y. (2017). A critical role of presynaptic cadherin/catenin/p140Cap complexes in stabilizing spines and functional synapses in the neocortex. *Neuron* 94 1155.e8–1172.e8. 10.1016/j.neuron.2017.05.022 28641114

[B28] Liebermeister W. Noor E. Flamholz A. Davidi D. Bernhardt J. Milo R. (2014). Visual account of protein investment in cellular functions. *Proc. Natl. Acad. Sci. U.S.A.* 111 8488–8493. 10.1073/pnas.1314810111 24889604 PMC4060655

[B29] Mclean C. Xin H. Ian Simpson T. Douglas Armstrong J. (2016). Improved functional enrichment analysis of biological networks using scalable modularity based clustering. *J. Proteomics Bioinform.* 9:10.

[B30] Menche J. Sharma A. Kitsak M. Ghiassian S. D. Vidal M. Loscalzo J. (2015). Disease networks. Uncovering disease-disease relationships through the incomplete interactome. *Science* 347:1257601. 10.1126/science.1257601 25700523 PMC4435741

[B31] Muller W. J. Sinn E. Pattengale P. K. Wallace R. Leder P. (1988). Single-step induction of mammary adenocarcinoma in transgenic mice bearing the activated c-neu oncogene. *Cell* 54 105–115. 10.1016/0092-8674(88)90184-5 2898299

[B32] Musen M. A. Noy N. F. Shah N. H. Whetzel P. L. Chute C. G. Story M. A. (2012). The national center for biomedical ontology. *J. Am. Med. Inform. Assoc.* 19 190–195. 22081220 10.1136/amiajnl-2011-000523 PMC3277625

[B33] Najafi M. McMenamin B. W. Simon J. Z. Pessoa L. (2016). Overlapping communities reveal rich structure in large-scale brain networks during rest and task conditions. *Neuroimage* 135 92–106. 10.1016/j.neuroimage.2016.04.054 27129758 PMC4915991

[B34] Nepusz T. Petroczi A. Negyessy L. Bazso F. (2008). Fuzzy communities and the concept of bridgeness in complex networks. *Phys. Rev. E* 77:016107. 18351915 10.1103/PhysRevE.77.016107

[B35] Pons P. Latapy M. (2006). Computing communities in large networks using random walks. *J. Graph Algorithms Appl.* 10 191–218. 10.7155/jgaa.00124

[B36] Reichardt J. Bornholdt S. (2006). Statistical mechanics of community detection. *Phys. Rev. E* 74:016110. 10.1103/PhysRevE.74.016110 16907154

[B37] Repetto D. Aramu S. Boeri Erba E. Sharma N. Grasso S. Russo I. (2013). Mapping of p140Cap phosphorylation sites: the EPLYA and EGLYA motifs have a key role in tyrosine phosphorylation and Csk binding, and are substrates of the Abl kinase. *PLoS One* 8:e54931. 10.1371/journal.pone.0054931 23383002 PMC3561454

[B38] Repetto D. Camera P. Melani R. Morello N. Russo I. Calcagno E. (2014). p140Cap regulates memory and synaptic plasticity through Src-mediated and citron-N-mediated actin reorganization. *J. Neurosci.* 34 1542–1553. 10.1523/JNEUROSCI.2341-13.2014 24453341 PMC6705312

[B39] Rovero S. Amici A. Di Carlo E. Bei R. Nanni P. Quaglino E. (2000). DNA vaccination against rat her-2/Neu p185 more effectively inhibits carcinogenesis than transplantable carcinomas in transgenic BALB/c mice. *J. Immunol.* 165 5133–5142. 10.4049/jimmunol.165.9.5133 11046045

[B40] Salwinski L. Miller C. S. Smith A. J. Pettit F. K. Bowie J. U. Eisenberg D. (2004). The database of interacting proteins: 2004 update. *Nucleic Acids Res.* 32 D449–D451. 14681454 10.1093/nar/gkh086 PMC308820

[B41] Schriml L. M. Arze C. Nadendla S. Chang Y. W. Mazaitis M. Felix V. (2012). Disease ontology: a backbone for disease semantic integration. *Nucleic Acids Res.* 40 D940–D946. 10.1093/nar/gkr972 22080554 PMC3245088

[B42] Simpson T. I. Armstrong J. D. Jarman A. P. (2010). Merged consensus clustering to assess and improve class discovery with microarray data. *BMC Bioinformatics* 11:590. 10.1186/1471-2105-11-590 21129181 PMC3002369

[B43] Tomasoni R. Repetto D. Morini R. Elia C. Gardoni F. Di Luca M. (2013). SNAP-25 regulates spine formation through postsynaptic binding to p140Cap. *Nat. Commun.* 4:2136. 10.1038/ncomms3136 23868368

[B44] Traag V. A. Bruggeman J. (2009). Community detection in networks with positive and negative links. *Physical. Rev. E* 80 036115. 19905188 10.1103/PhysRevE.80.036115

[B45] Tyanova S. Temu T. Cox J. (2016). The MaxQuant computational platform for mass spectrometry-based shotgun proteomics. *Nat. Protoc.* 11 2301–2319. 10.1038/nprot.2016.136 27809316

[B46] Vizcaino J. A. Csordas A. Del-Toro N. Dianes J. A. Griss J. Lavidas I. (2016). 2016 update of the PRIDE database and its related tools. *Nucleic Acids Res.* 44 D447–D456. 10.1093/nar/gkv1145 26527722 PMC4702828

[B47] Whetzel P. L. Noy N. F. Shah N. H. Alexander P. R. Nyulas C. Tudorache T. (2011). BioPortal: enhanced functionality via new web services from the national center for biomedical ontology to access and use ontologies in software applications. *Nucleic Acids Res.* 39 W541–W545. 10.1093/nar/gkr469 21672956 PMC3125807

[B48] Wieczorek S. Combes F. Lazar C. Giai Gianetto Q. Gatto L. Dorffer A. (2017). DAPAR & ProStaR: software to perform statistical analyses in quantitative discovery proteomics. *Bioinformatics* 33 135–136. 27605098 10.1093/bioinformatics/btw580 PMC5408771

[B49] Yu G. Wang L. G. Han Y. He Q. Y. (2012). clusterProfiler: an R package for comparing biological themes among gene clusters. *OMICS* 16 284–287. 10.1089/omi.2011.0118 22455463 PMC3339379

