# Supplementary material for: Dissecting the Shared and Context-Dependent Pathways Mediated by the p140Cap Adaptor Protein in Cancer and in Neurons
Source: Front Cell Dev Biol. 2019 Oct 15;7:222. doi: 10.3389/fcell.2019.00222 (PMC6803390; doi:10.3389/fcell.2019.00222)
Supplement: FIGURE S1 — In vitro characterization of p140Cap-TuBo cell line. [file Data_Sheet_1.PDF]

Legend to Suppl. Figure 1.

In vitro characterization of p140Cap-TuBo cell line.

A) $5x10^3$ Mock and p140Cap cells were plated in 96-wells plate and the cell viability was evaluated by MTT assay. Three independent experiments were performed. Differences in cell viability were evaluated using two-way ANOVA followed by Bonferroni multiple comparison post hoc tests (***p < 0.001).

B) $5x10^4$ Mock and p140 cells were plated in 6-wells plate and ability to growth in soft agar and form colonies was evaluated at day15. Two parameters were analysed and reported in histograms (mean ± SEM) to compare Mock and p140 cells using unpaired t-tests (*p < 0.05).
