# Supplementary material for: Dissecting the Shared and Context-Dependent Pathways Mediated by the p140Cap Adaptor Protein in Cancer and in Neurons
Source: Front Cell Dev Biol. 2019 Oct 15;7:222. doi: 10.3389/fcell.2019.00222 (PMC6803390; doi:10.3389/fcell.2019.00222)
Supplement: FIGURE S2 — (A–C) Panels represent three distinct TUBO cell extracts that were immunoprecipitated using p140Cap monoclonal antibody. A: exp 1, B: exp 2; C: exp 3. The immunoprecipitates and the corresponding cell extracts (30 micrograms) were run on a 4–15% SDSPAGE and the nitrocellulose membranes were cut according to the molecular weight, in order to decorate the upper part with the p140Cap antibodies and the lower part with the Tubulin antibodies for loading controls. On the left, we show the merge between the colorimetric WB with the chemiluminescent WB, obtained at the ChemiDoc Imaging System from BIO RAD. Membranes were all exposed for 30 sec. [file Data_Sheet_2.PDF]

Supplementary Figure 2

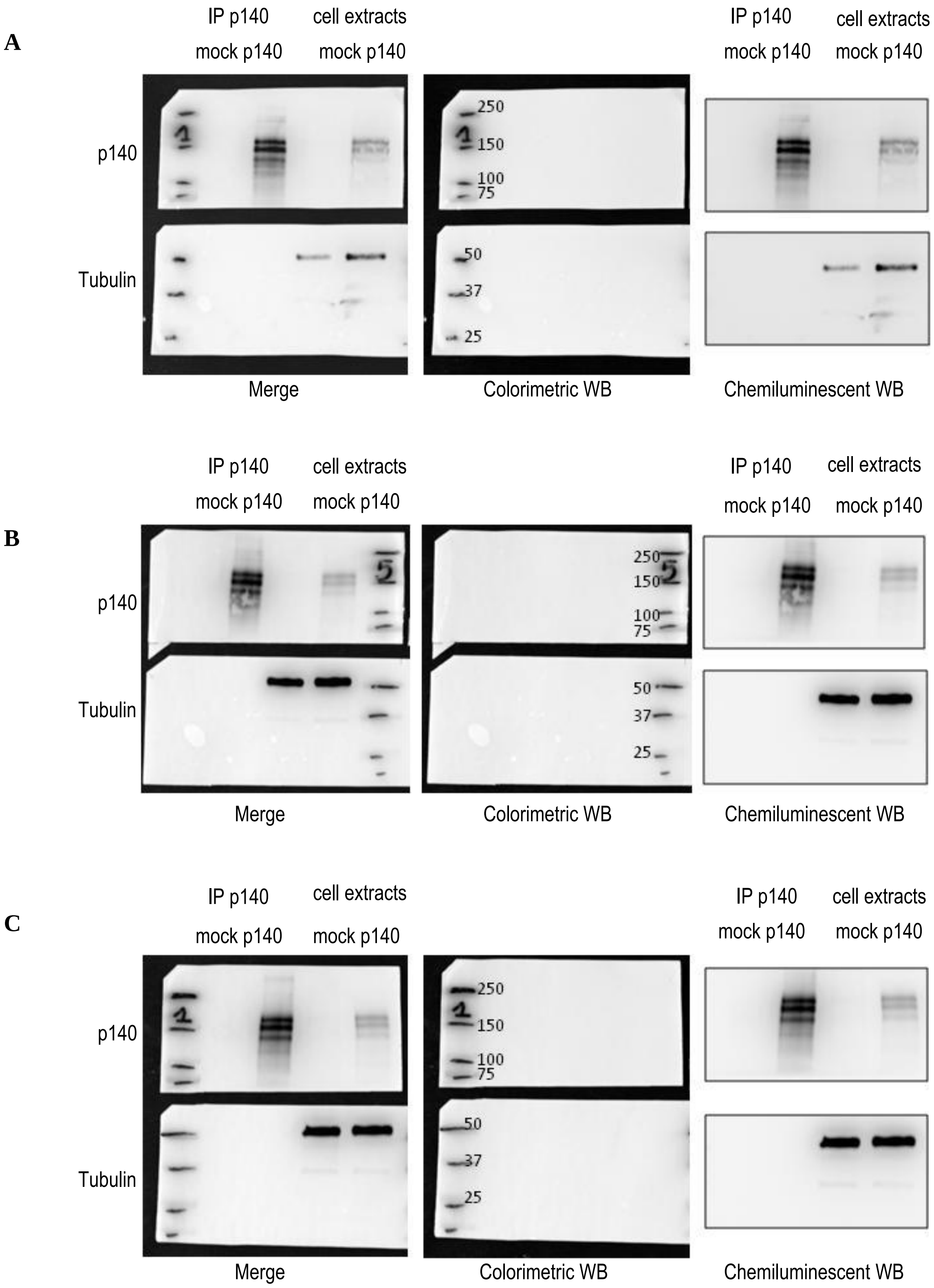

A, B, C panels represent three distinct TUBO cell extracts that were immunoprecipitated using p140Cap monoclonal antibody. A: exp 1, B: exp 2; C: exp 3.

The immunoprecipitates and the corresponding cell extracts (30 micrograms) were run on a 4-15% SDS-PAGE and the nitrocellulose membranes were cut according to the molecular weight, in order to decorate the upper part with the p140Cap antibodies and the lower part with the Tubulin antibodies for loading controls. On the left, we show the merge between the colorimetric WB with the chemiluminescent WB, obtained at the ChemiDoc Imaging System from BIO RAD. Membranes were all exposed for 30 sec.
