# Supplementary figures and images for: Dissecting the Shared and Context-Dependent Pathways Mediated by the p140Cap Adaptor Protein in Cancer and in Neurons
Source: Front Cell Dev Biol. 2019 Oct 15;7:222. doi: 10.3389/fcell.2019.00222 (PMC6803390; doi:10.3389/fcell.2019.00222)

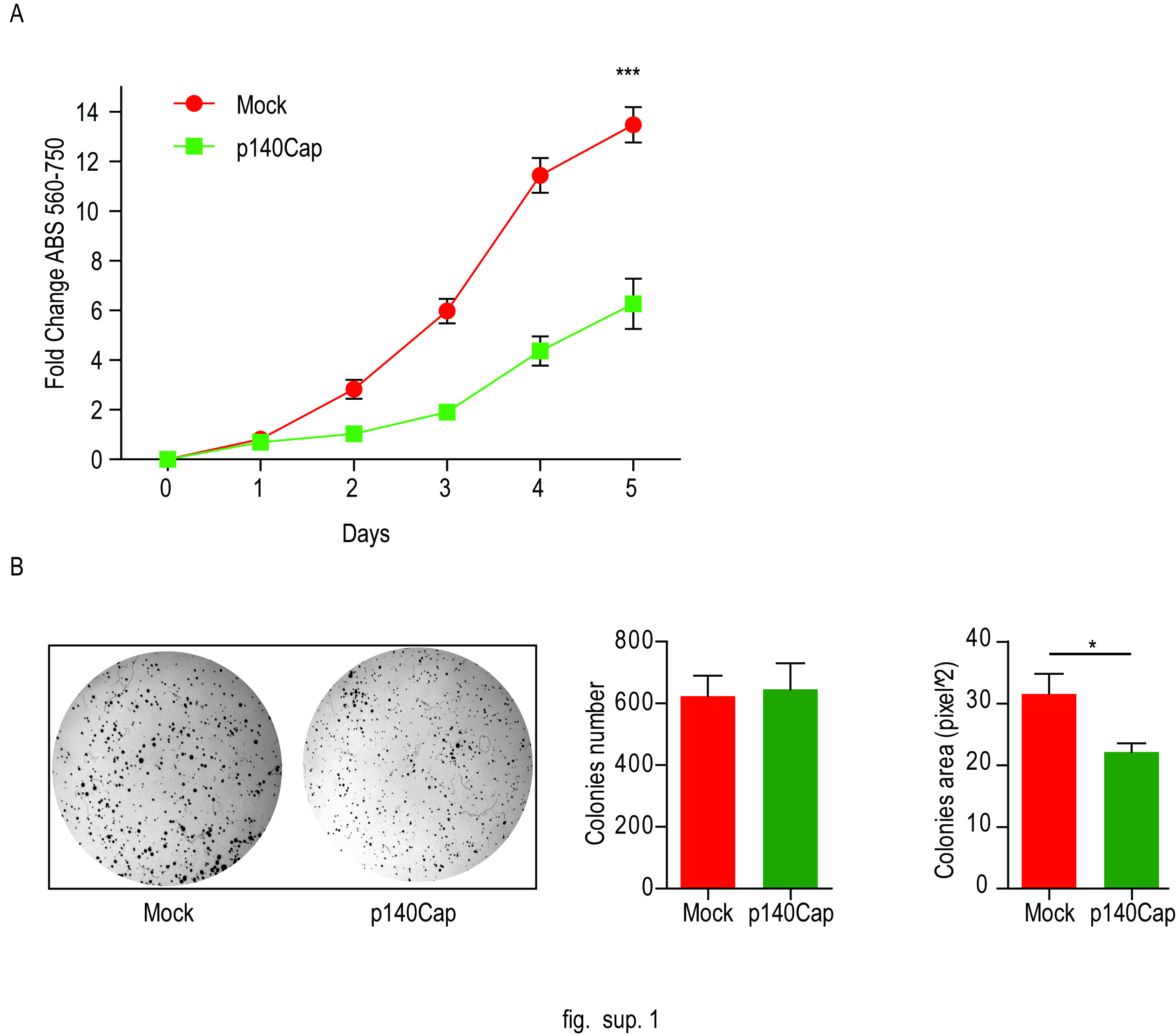

Supplement: Supplementary file 9 [file Image_1.TIF]
